# Laser Peripheral Iridotomy Curriculum: Lecture and Simulation Practical

**DOI:** 10.15766/mep_2374-8265.10903

**Published:** 2020-05-27

**Authors:** Joanne C. Wen, Kasra A. Rezaei, Deborah L. Lam

**Affiliations:** 1 Associate Professor, Department of Ophthalmology, University of Washington School of Medicine; 2 Assistant Professor, Department of Ophthalmology, University of Washington School of Medicine; 3 Associate Professor, Department of Ophthalmology, University of Washington School of Medicine

**Keywords:** Laser Peripheral Iridotomy, Nd:YAG, Ophthalmology, Clinical/Procedural Skills Training, Simulation

## Abstract

**Introduction:**

Approximately 20 million people worldwide are affected by primary angle closure glaucoma, which is often treated with a laser peripheral iridotomy (LPI). In the United States, at least 60,000 to 80,000 LPIs are performed annually. However, complications can arise from improperly performed LPIs. While the Accreditation Council for Graduate Medical Education requires that all ophthalmology residents perform at least four primary LPIs prior to graduating, formal training is often lacking. In an effort to standardize LPI teaching, an LPI lecture curriculum and skills practice session were introduced.

**Methods:**

A lecture and wet-lab curriculum was developed at the University of Washington to formally teach first-year ophthalmology residents the indications and techniques for LPI. Pre- and postcurriculum knowledge was tested, and LPI performance was assessed by comparing pre- and postcurriculum total number of shots and time needed to successfully complete an LPI on a commercially available model eye.

**Results:**

The course was highly rated by 10 residents (all PGY 2), with an increase in pre- versus posttest scores, an improvement in LPI performance metrics, and an increase in pre- versus postcurriculum scores for the three survey questions regarding curriculum objectives.

**Discussion:**

This course improved learner knowledge and confidence in performing LPI. Test scores improved following the course, as did self-assessed confidence levels of the residents. Residents made a number of positive comments about the course. We plan to continue holding this training session every year at our institution.

## Educational Objectives

By the end of this session, learners will be able to:

1. List the indications for laser peripheral iridotomy (LPI).
2. Accurately explain the LPI procedure and postprocedure management to patients and obtain informed consent.
3. Become proficient in the technical skills involved with performing safe and effective LPIs.

## Introduction

Glaucoma is a chronic, progressive optic neuropathy that is a leading cause of irreversible blindness. By 2020, glaucoma is projected to affect close to 80 million people worldwide. Approximately one-fourth of cases are due to a subtype of glaucoma called primary angle closure glaucoma (PACG).^1^ Laser peripheral iridotomy (LPI) is commonly used as a treatment for PACG and primary angle closure as well as a preventative procedure in patients who are primary angle closure suspects.^2^ In the United States, 60,000 to 80,000 LPIs are performed each year on Medicare recipients.^3^ Given the prevalence of this disease and the high likelihood that ophthalmologists will need to perform an LPI during their careers, the Accreditation Council for Graduate Medical Education expects all ophthalmology residents to have performed at least four primary LPIs prior to graduating. However, formal teaching in proper indications and techniques for LPIs is often lacking in many institutions. While LPIs are relatively low-risk, complications can arise from them including bleeding, prolonged inflammation, intraocular pressure elevations, lens or cornea damage, and dysphotopsias.^4,5^ The University of Washington recently reviewed all resident-performed LPIs over a 5-year period and found that while total energy use and complication rates were comparable to attending-performed LPIs in the literature, there was a higher incidence of repeat laser to reopen or enlarge peripheral iridotomies.^6^ In an effort to standardize LPI teaching and decrease the incidence of repeat LPI, as well as to help maintain minimal complication rates, an LPI curriculum including a lecture and a skills practice session was introduced.

This curriculum was implemented at the University of Washington in 2018 and has been held annually. The target audience was primarily first-year ophthalmology residents, although residents and fellows at any level could benefit from the curriculum. The curriculum consisted of two sessions with an interval period for independent practice. During the first session, there was a pretest, followed by a lecture reviewing indications for LPI, risks and benefits, pre- and postprocedure management, and an overview of the LPI procedure. After the lecture, the learners participated in a skills practice session and took an initial LPI assessment. The learners were each given a model eye to practice with on their own time, and a later session was scheduled for a posttest.

## Methods

### Development

The residency program at the University of Washington included weekly time dedicated to didactics, including lectures and wet labs. Our LPI curriculum was implemented during two of these scheduled didactic sessions approximately 6 months into the academic year. The target learners were first-year ophthalmology residents who had a basic knowledge of ocular anatomy and pathology, although second- and third-year residents were encouraged to participate in the lecture. The facilitator was a glaucoma specialist who had significant experience at performing LPIs.

### Equipment/Environment

**Figure 1.**
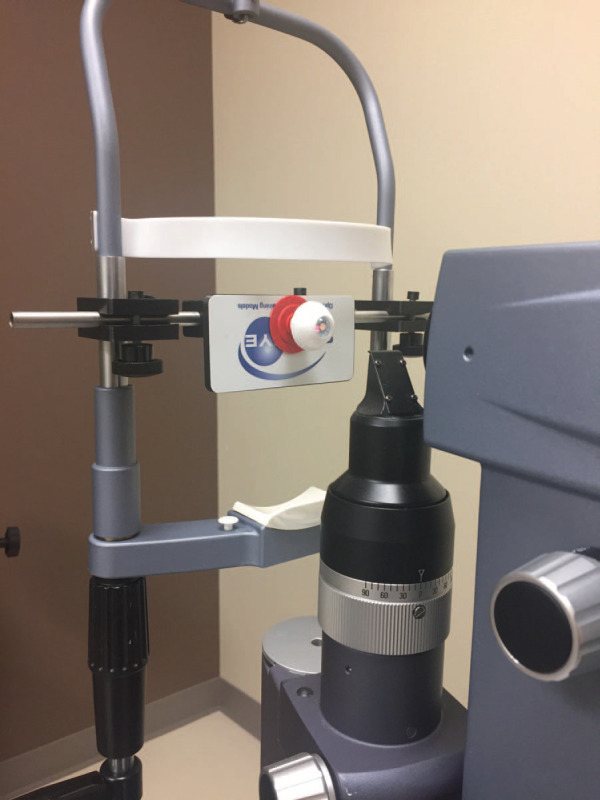
Example of laser simulation setup with the SimulEYE slit lamp holder and SimulEYE LPI model eye.

The curriculum required the following:

• Access to a conference or lecture room with a projector to give the lecture.
• Access to an Nd:YAG laser used to perform LPIs.
• Laser safety goggles of appropriate wavelength for the Nd:YAG laser.
• SimulEYE LPI models (www.guldenophthalmics.com, Product Number: 17028, $100 for two eyes):
  ○ For each SimulEYE LPI model eye, four LPIs could be completed.
  ○ Course facilitators had to order enough model eyes for learners to each complete one LPI during the initial LPI assessment and one LPI during the posttest, as well as one eye for each learner to practice on between sessions.
  ○ The SimulEYE LPI model eye had to be filled with water prior to mounting on the slit lamp holder (Figure 1).
• SimulEYE slit lamp holder (www.guldenophthalmics.com, Product Number: 17030, $100 for one):
  ○ This had to be mounted on the Nd:YAG laser during the setup for the curriculum (Figure 1).

Alternatively, a noncommercial LPI model eye could be constructed with materials described by Simpson, Schweitzer, and Johnson.^7^

### Personnel

Given the relatively small size of ophthalmology resident classes (at the University of Washington, five residents), a single instructor gave the lecture and supervised the residents as they practiced on the LPI model. The instructor was experienced in performing LPIs.

### Implementation

The curriculum was held over two sessions. The first was a 1-hour period that included a pretest, the lecture and skills practice session, and an initial LPI assessment. The second session was a 30-minute period for the posttest. The curriculum schedule was as follows:

• Session 1:
  ○ Pretest (10 minutes; Appendix A).
  ○ Lecture (20 minutes; Appendix B).
  ○ Skills demonstration (15 minutes).
  ○ Initial LPI assessment (15 minutes; Appendix C).
• Session 2:
  ○ Final LPI assessment and posttest (30 minutes; Appendices D and E).

### Session 1

The Nd:YAG laser was readily accessible so that immediately following the lecture, the instructor could provide instruction on the laser to the learner to demonstrate basic setup. Because lasers manufactured by different companies may differ from the lasers used in this curriculum, instructors at other institutions should familiarize themselves with their own lasers prior to implementing the curriculum.

The instructor had set up target materials at the laser prior to initiating the skills practice session. The SimulEYE LPI model eye was filled with water per manufacturer instruction, being careful to avoid air bubbles in the anterior portion of the model. The SimulEYE slit lamp holder was mounted to the laser per manufacturer instruction and the SimulEYE LPI model eye suctioned to the slit lamp holder (Figure 1). Laser safety goggles were readily available.

Future instructors should review the lecture notes that accompany the lecture slides prior to the session to ensure adequate understanding and familiarity with the lecture material (Appendix B). Sufficient numbers of pretests (Appendix A) should be printed for the class. A sufficient number of model eyes for independent practice should be available at the end of this session.

At the beginning of the session, pretests (Appendix A) were distributed, and learners had 10 minutes to complete the test. This was followed by the lecture (Appendix B) for approximately 20 minutes. Instructor and learners then proceeded to the area where the Nd:YAG laser was set up. There, the instructor demonstrated and reviewed the following:

• Ensured proper signs or other methods for notifying others that a laser was in progress were prominently displayed.
• Ensured all observers were wearing proper safety goggles.
• Demonstrated how to turn on the laser.
• Demonstrated how to adjust slit beam and aiming beam illumination intensity.
• Demonstrated how to adjust slit beam size.
• Demonstrated how to align the target with the slit lamp beam and aiming beam.
• Demonstrated optimal appearance of aiming the beam when properly focused.
• Demonstrated how to apply the coupling gel to the LPI lens.

Then, the learner practiced doing the above steps under direct supervision.

For the initial LPI assessment (Appendix C), the learner performed an LPI on the LPI model under direct supervision (energy set at 5.0 mJ). Total number of shots and total time from laser lens contact to LPI completion were documented. Note: Air bubbles were noted to form, which could have obstructed the superior portion of the LPI model, so we recommend avoiding the superior location for LPI practice. The model can be rotated such that all LPI locations within it can be positioned along the horizontal meridian.

Each learner was given an LPI model eye for independent practice.

### Session 2

The instructor prepared the laser materials and models as previously described for Session 1. Learners were taken individually to the Nd:YAG laser and asked to demonstrate all the elements on the final LPI assessment (Appendix D). Next, learners were asked to perform an LPI on the LPI model, and total number of shots and total time from laser lens contact to LPI completion were documented. Learners were then given the LPI posttest (Appendix E). Instructors used the pre-/posttest answer key (Appendix F) to grade the tests.

### Assessment

Curriculum effectiveness was evaluated in a number of ways. The pre- and posttests assessed improvement in the learners’ LPI-related fund of knowledge. The final LPI assessment was a checklist of tasks that we felt represented all the functions of the Nd:YAG laser a learner should know to safely operate the machine. LPI performance improvement was assessed by comparing pre- and postcurriculum total number of shots needed to complete an LPI and time to complete an LPI. A study by Kam, Zepeda, Ding, and Wen demonstrated decreasing power usage among residents performing LPI procedures with increasing resident training stage, suggesting that decreased total power to complete an LPI might represent increasing procedural proficiency.^6^ Therefore, assessing total number of laser shots in this curriculum was a way to measure procedural proficiency. Lastly, learners were asked to rate pre- and postcurriculum confidence scores for the three learning objectives and to provide feedback on the curriculum. Pre- and posttests, metrics, and ratings were compared with a Wilcoxon signed rank test. A *p* value less than .05 was considered statistically significant.

## Results

**Figure 2.**
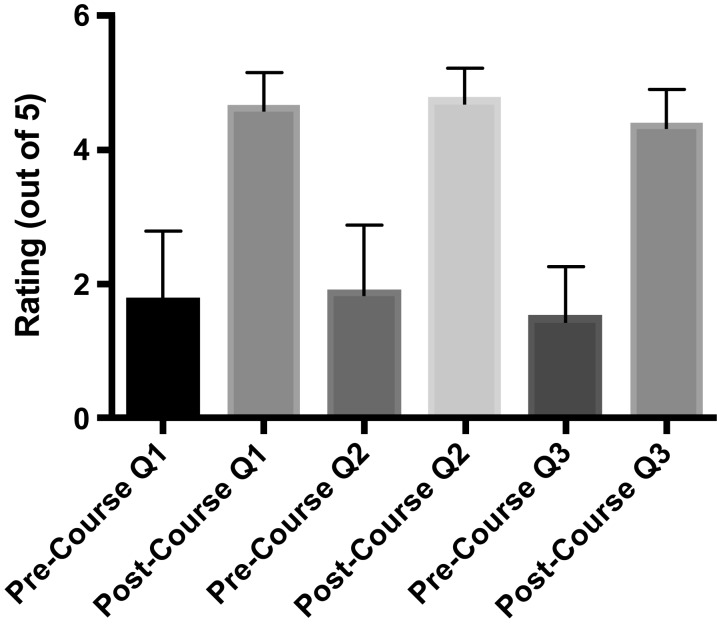
Pre- versus posttest scores. Means and standard deviations are shown (*p* = .008).

**Figure 3.**
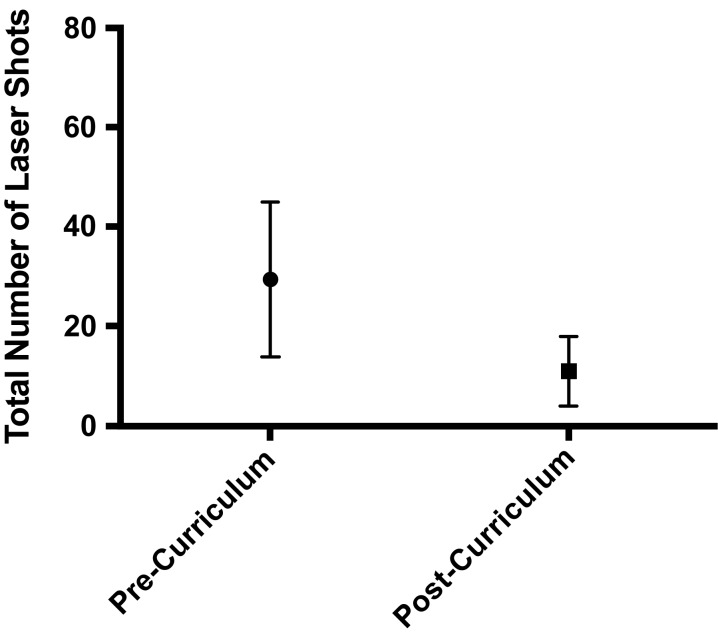
Comparison of total number of laser shots needed to complete a laser peripheral iridotomy pre- and postcurriculum. Means and standard deviations are shown (*p* = .02).

**Figure 4.**
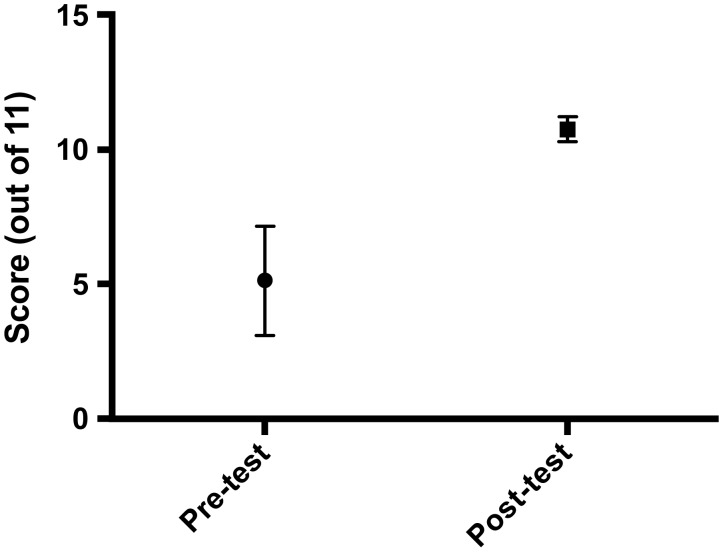
Pre- versus postcourse ratings for the three course objectives. Means and standard deviations are shown.

There were five residents per ophthalmology residency class at the University of Washington. Since the curriculum's introduction in 2018, 10 residents had completed it. In terms of fund of knowledge, there was a significant increase in pre- versus posttest scores following the course (means of 5.1 ± 2.0 vs. 10.8 ± 0.5, respectively, perfect score = 11, *p* = .008; Figure 2). All learners correctly performed all tasks on the final LPI assessment. With respect to LPI performance metrics, there was a significant decrease in the total number of laser shots needed to complete an LPI (mean of 29.4 ± 15.6 shots decreased to a mean of 10.9 ± 7.0 shots, *p* = .02; Figure 3) although total time to complete the LPI was not significantly changed (mean of 90.5 ± 31.2 seconds vs. mean of 89.3 ± 33.5 seconds, *p* = 1.0). There was an increase in pre- versus postcurriculum scores for the three survey questions regarding curriculum objectives (all on a scale of 1–5, with 1 = *not comfortable at all* and 5 = *very comfortable*; Figure 4):

• Survey question 1: How comfortable are you with knowing the indications for performing an LPI?
  ○ Pretest mean score of 1.8 ± 1.0 versus posttest mean score of 4.6 ± 0.5 (*p* < .01).
• Survey question 2: How comfortable are you with discussing the risks and benefits of an LPI with a patient?
  ○ Pretest mean score of 1.9 ± 1.0 versus posttest mean score of 4.8 ± 0.5 (*p* < .01).
• Survey question 3: How comfortable are you with performing an LPI?
  ○ Pretest mean score of 1.5 ± 0.8 versus posttest mean score of 4.4 ± 0.5 (*p* < .01).

The course was highly rated, with the average response to the question “How would you rate this course overall?” being a 4.9 out of 5 (90% response rate; 5-point scale, with 1 = *poor* and 5 = *outstanding*).

Three participants provided qualitative feedback on the course:

• “I thought it was extremely helpful.”
• “Super good lecture!”
• “Very valuable, thank you so much!”

## Discussion

To teach residents LPI indications, risks, and proper LPI technique, we developed this formalized curriculum with objective measures for assessing LPI knowledge and the LPI procedure on a simulation model. Learners were given pre- and posttests to evaluate improvements in LPI knowledge, and total number of laser shots to complete an LPI and total procedural time were evaluated at the beginning and end of the curriculum. Overall, we found that our curriculum improved learner knowledge about LPI, learner ability to perform a proper LPI on the practice model, and learner self-assessed confidence in the three objectives of the curriculum.

We chose a plastic eye model as it allowed the use of the focusing lens and coupling gel to best simulate real-life conditions. The use of a similar model was described in a study by Simpson and colleagues, who designed a model eye with artificial tissues to simulate common ophthalmic laser procedures including LPI, laser capsulotomy, and laser retinopexy.^7^ They compared inexperienced (PGY 2) with experienced (PGY 4) ophthalmology residents and found a nonsignificant trend towards decreased number of shots needed to complete an LPI and no difference in total time needed to complete the LPI.^7^ They attributed the nonsignificant difference in total number of laser shots to the high-power setting (9.0 mJ) they used in their simulation, which may have overcompensated for poor technique and therefore masked differences. Our simulation used a much lower energy setting of 5.0 mJ, and we did find a significant difference in total number of laser shots, supporting Simpson and colleagues’ hypothesis that at lower energy levels, the importance of aiming and focusing technique may be more apparent.

Identifying an appropriate model to simulate procedures is crucial. Recent improvements in ophthalmologic simulation models have increased teaching options for ophthalmic laser education. Notably, we initially developed an LPI curriculum at the University of Washington in 2016 that was very similar to the current one except that model eye options were limited and so residents practiced lasering a tomato (this was recommended by the laser manufacturer). In this resident cohort, there was also an increase in pre- versus postcurriculum test scores following the course (means of 6.8 ± 0.4 vs. 10.6 ± 0.4, respectively), as well as an increase in all postcurriculum survey questions. However, the postcurriculum score for survey question 3 (“How comfortable are you with performing an LPI?”) achieved a mean of only 3.8 ± 0.8. We attributed this relatively low score to the less realistic practice model and were pleased to see that learners of the current curriculum had a mean of 4.4 ± 0.5 on that same survey question, suggesting that this practice model improved learner performance confidence.

Most ophthalmology residency programs should be able to implement this curriculum using the suggested models and materials described. However, if these models and materials are cost prohibitive, the previously mentioned model by Simpson and colleagues may provide a lower-cost alternative.^7^ In their study, the laser model was constructed with materials readily available at most craft stores, including a clear plastic sphere, white paint, a microscope slide, and blue tissue paper, for a total cost of approximately $10.^7^ For programs that are unable to purchase or create the above models, a practice target such as a tomato, while limited in the ability to simulate using a laser focusing lens, is still useful for demonstrating laser setup and focusing on a target. As previously mentioned, our original curriculum given in 2016 used a tomato for practice, and learners still demonstrated improved postcurriculum test scores with increased confidence (assessed by rating “How comfortable are you with performing an LPI?” on a 5-point scale) in performing the LPI procedure (mean of 2.8 ± 0.8 precurriculum vs. mean of 3.8 ± 0.8 postcurriculum). Therefore, the implementation of this curriculum even with a less realistic model can still be very useful for teaching the fundamentals of the LPI procedure.

The optimal location for LPI placement within the eye is controversial, with evidence to support the temporal or superior location. Vera and colleagues randomized each eye of patients who needed bilateral LPIs to either superior or temporal LPI positions and found a significantly greater incidence of new-onset linear dysphotopsias in eyes with a superior LPI.^8^ Conversely, a study by Srinivasan and colleagues randomized both eyes of patients to either superior or temporal/nasal LPI locations and did not find a significant difference in reported new-onset dysphotopsias.^9^ Our institutional preference is to place them in the temporal location; however, given the lack of clear evidence, people who implement this curriculum may choose to recommend either location.

Of note, many of our senior residents chose to participate in the lecture portion of this curriculum. While most had previously performed LPIs, many commented that they found the lecture material informative and useful. Specifically, clarifying the importance of laser safety goggles that cover the appropriate wavelength for the laser being used was cited as particularly helpful. Also, specifying that the LPI size should be at least 150–200 μm was informative for nearly all learners as this was a commonly missed question on the pretest.

A retrospective study conducted at the University of Washington looking at the efficacy and safety of resident-performed LPI found that energy use decreased significantly with increasing resident training while complication rates were low and did not change significantly among the three classes.^2^

Furthermore, energy use and complication rates were comparable to what had been reported in the literature for attending-performed LPI procedures. Decreasing energy use may be a sign of improving procedural proficiency. In this curriculum, we found a significant decrease in the total number of shots (and correspondingly total energy) needed to complete the LPI, though total time to complete the LPI did not change. It appears that learners still dedicated the same amount of time for the procedure but had more effective laser technique after the curriculum. As we continue to offer this course annually, we hope to improve LPI procedural proficiency at an earlier stage in training, which will hopefully be reflected in lower total energy usage much sooner in training.

There are a few limitations of this simulation model. One limitation is that learners are unable to practice LPI using the argon laser. Additionally, when performing LPI on a patient, a gush of fluid and posterior pigmented epithelium can be seen once the iris is fully penetrated. This is not seen with the current model. We did consider using enucleated porcine eyes, as these are commonly used for practice of other ophthalmic procedures, but the biological hazard risks of contaminating lasers that are also in clinical use was too great. Models for ophthalmic procedure simulation are in constant development, so in the future, a model that allows practice with an argon laser and better simulates the visual feedback of a completed LPI may become available.

Our methods for assessing improvement were limited to the classroom setting and did not include skill assessments in actual clinical settings. While the classroom setting provided objective end points for evaluation, such as number of laser shots and time to LPI completion, additional assessments of LPI proficiency in the clinic are the goal. Additional assessments could include having the residents maintain a detailed log of their first five to 10 LPI procedures where number of shots, total energy usage, and complications are recorded. These logs could be reviewed by an attending ophthalmologist and feedback provided to the residents. At our institution, once this curriculum has been given for a few consecutive years, it is our hope to conduct a follow-up study to the one published by Kam and colleagues^6^ to assess for improvements in LPI proficiency.

Given the high average 4.9 out of 5 rating by our learners, we feel that this course is valuable in improving learner knowledge and confidence in performing LPIs. We plan on continuing this training session every year with an emphasis on ensuring participation from the newest residents and encouraging more senior residents to consider refresher course participation.

## Appendices

Pretest.docxLecture and Notes.pptxInitial LPI Assessment.docxFinal LPI Assessment.docxPosttest.docxPre- & Posttest Answers.docx
*All appendices are peer reviewed as integral parts of the Original Publication.*
