# Supplementary material for: Laser Peripheral Iridotomy Curriculum: Lecture and Simulation Practical
Source: MedEdPORTAL. 2020 May 27;16:10903. doi: 10.15766/mep_2374-8265.10903 (PMC7331967; doi:10.15766/mep_2374-8265.10903)
Supplement: Supplementary file 1 — Pretest.docxLecture and Notes.pptxInitial LPI Assessment.docxFinal LPI Assessment.docxPosttest.docxPre- & Posttest Answers.docx [file mep_2374-8265.10903-s001.zip › A. Pretest.docx]

1. Define the following terms according to the American Academy of Ophthalmology

Primary Angle Closure Suspect:

Primary Angle Closure:

Primary Angle Closure Glaucoma:

2. Which ethnic group as the highest incidence of angle closure?

a. Chinese

b. Inuit

c. Hispanics

d. African and African-derived

3. Benefits of using a laser iridotomy lens include all of the following EXCEPT:

a. Magnification of treatment area

b. Focuses energy on the iris

c. Increases energy density on cornea

d. Keeps eyelids open

4. List 4 potential complications following LPI

1.

2.

3.

4.

5. Where should the peripheral iridotomy be placed on the iris to minimize the risk of post-laser dysphotopsia?

a. superior

b. inferior

c. nasal

d. temporal

6. A peripheral iridotomy should be at least what size in diameter?

How comfortable are you with knowing the indications for performing a LPI?

Not comfortable at all 1 2 3 4 5 Very comfortable

How comfortable are you with discussing the risks and benefits of a LPI with a patient?

Not comfortable at all 1 2 3 4 5 Very comfortable

How comfortable are you with performing a LPI?

Not comfortable at all 1 2 3 4 5 Very comfortable
