# Supplementary material for: Laser Peripheral Iridotomy Curriculum: Lecture and Simulation Practical
Source: MedEdPORTAL. 2020 May 27;16:10903. doi: 10.15766/mep_2374-8265.10903 (PMC7331967; doi:10.15766/mep_2374-8265.10903)
Supplement: Supplementary file 1 — Pretest.docxLecture and Notes.pptxInitial LPI Assessment.docxFinal LPI Assessment.docxPosttest.docxPre- & Posttest Answers.docx [file mep_2374-8265.10903-s001.zip › B. Lecture and Notes.pptx]

## Slide 1
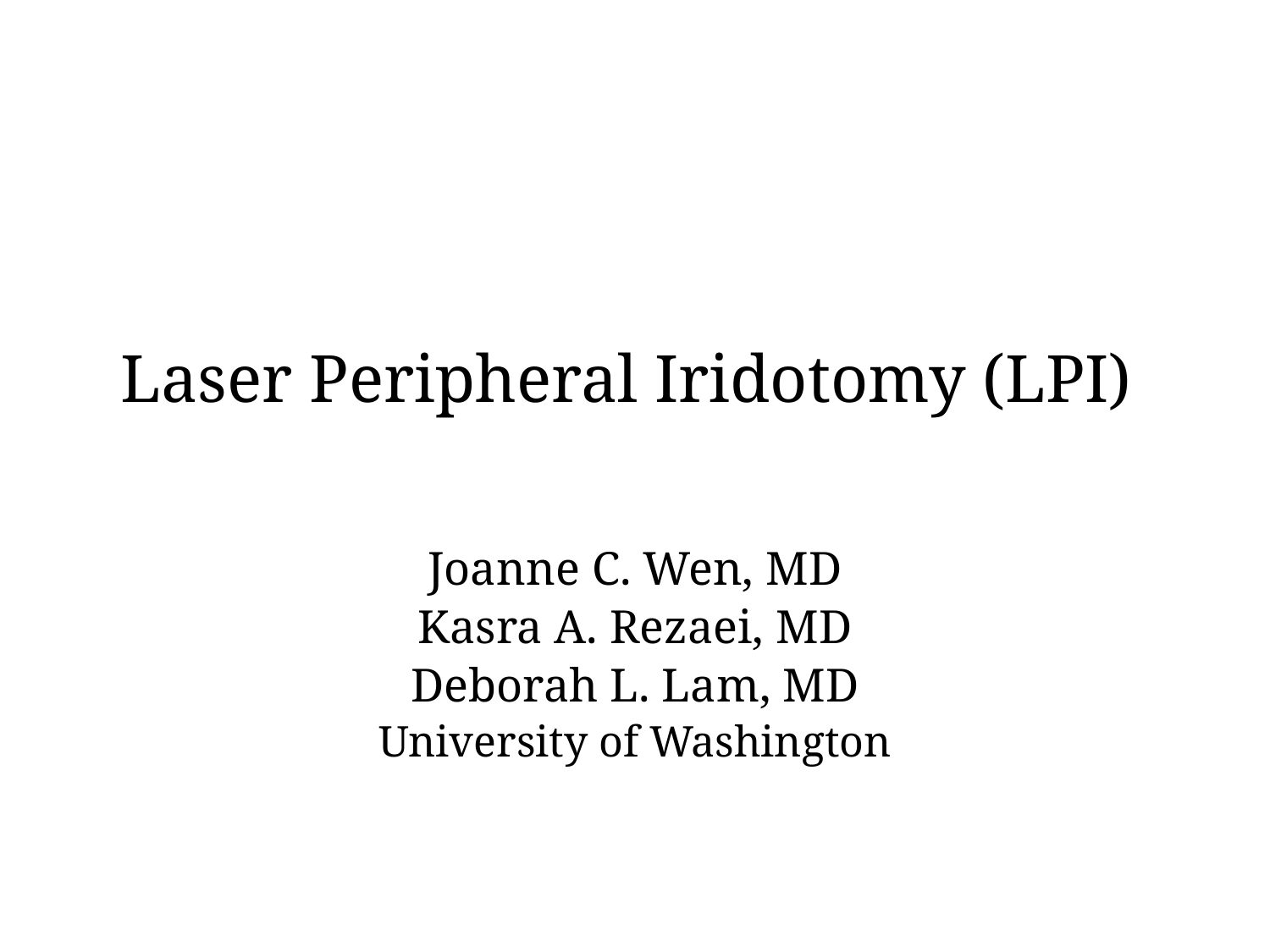

# Laser Peripheral Iridotomy (LPI)
Joanne C. Wen, MD
Kasra A. Rezaei, MD
Deborah L. Lam, MD
University of Washington

## Slide 2
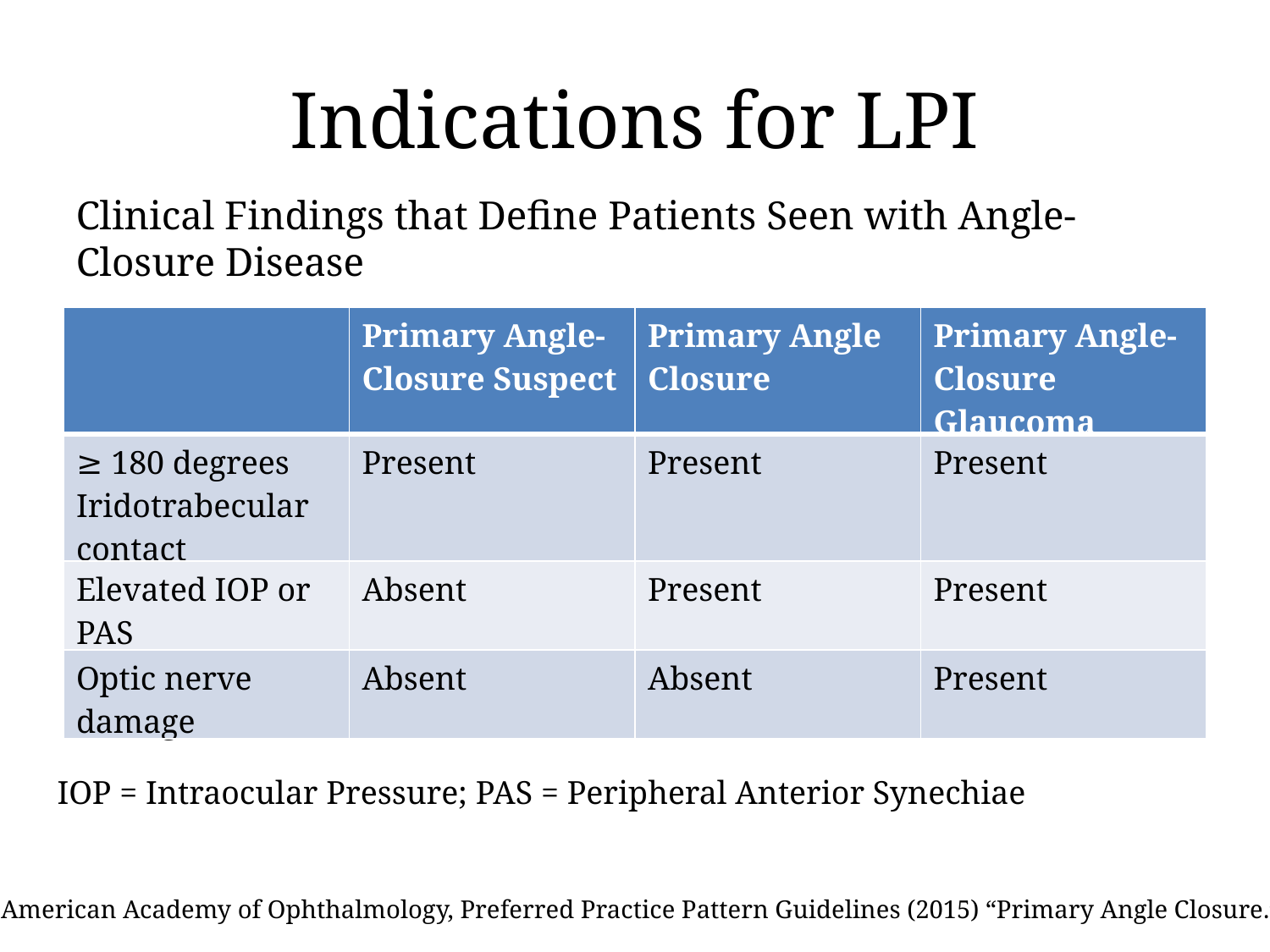

# Indications for LPI
Clinical Findings that Define Patients Seen with Angle-Closure Disease
| | Primary Angle-Closure Suspect | Primary Angle Closure | Primary Angle-Closure Glaucoma |
| --- | --- | --- | --- |
| ≥ 180 degrees Iridotrabecular contact | Present | Present | Present |
| Elevated IOP or PAS | Absent | Present | Present |
| Optic nerve damage | Absent | Absent | Present |
IOP = Intraocular Pressure; PAS = Peripheral Anterior Synechiae
American Academy of Ophthalmology, Preferred Practice Pattern Guidelines (2015) “Primary Angle Closure.”

## Slide 3
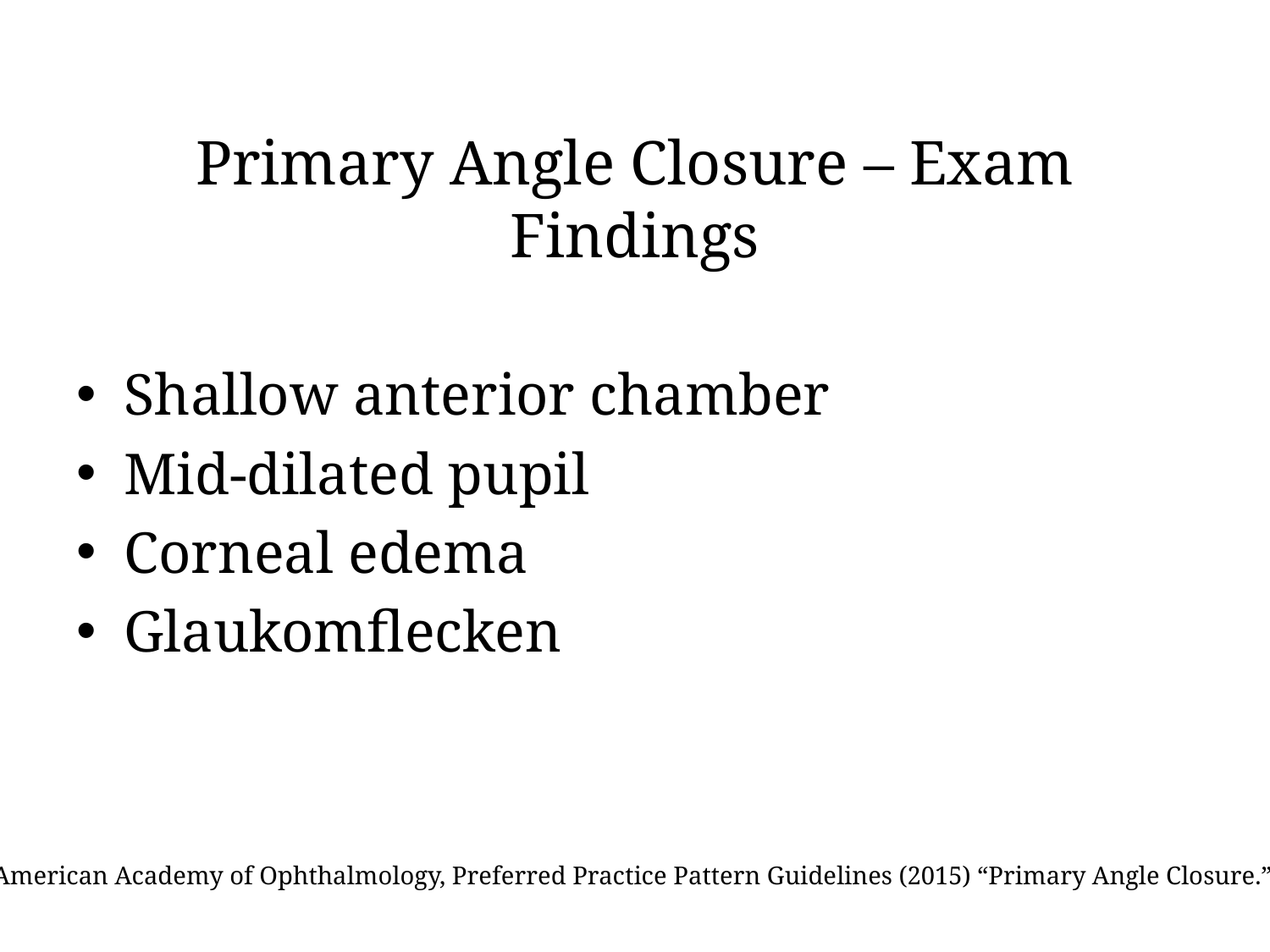

# Primary Angle Closure – Exam Findings
Shallow anterior chamber
Mid-dilated pupil
Corneal edema
Glaukomflecken
American Academy of Ophthalmology, Preferred Practice Pattern Guidelines (2015) “Primary Angle Closure.”

## Slide 4
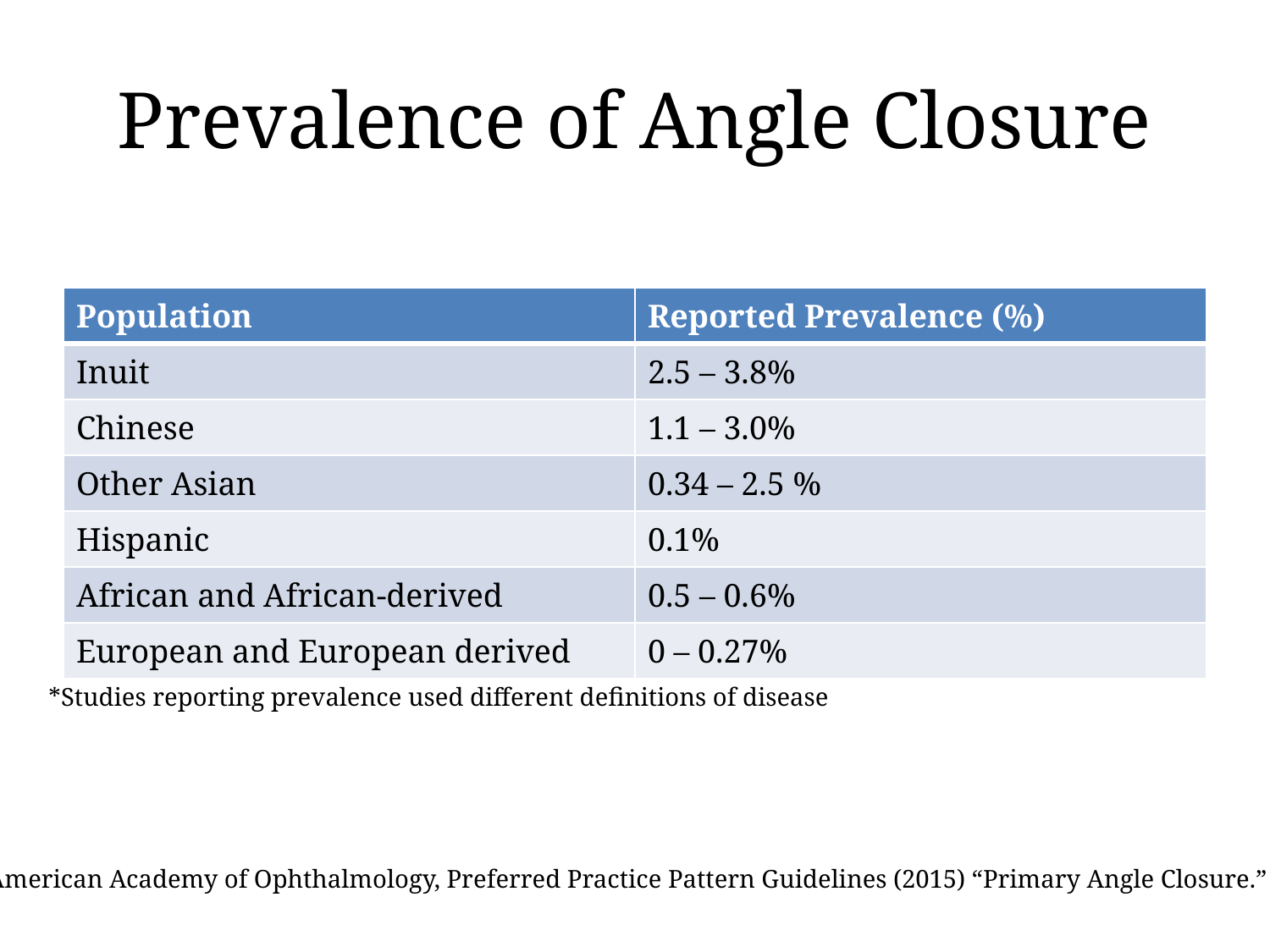

# Prevalence of Angle Closure
| Population | Reported Prevalence (%) |
| --- | --- |
| Inuit | 2.5 – 3.8% |
| Chinese | 1.1 – 3.0% |
| Other Asian | 0.34 – 2.5 % |
| Hispanic | 0.1% |
| African and African-derived | 0.5 – 0.6% |
| European and European derived | 0 – 0.27% |
*Studies reporting prevalence used different definitions of disease
American Academy of Ophthalmology, Preferred Practice Pattern Guidelines (2015) “Primary Angle Closure.”

## Slide 5
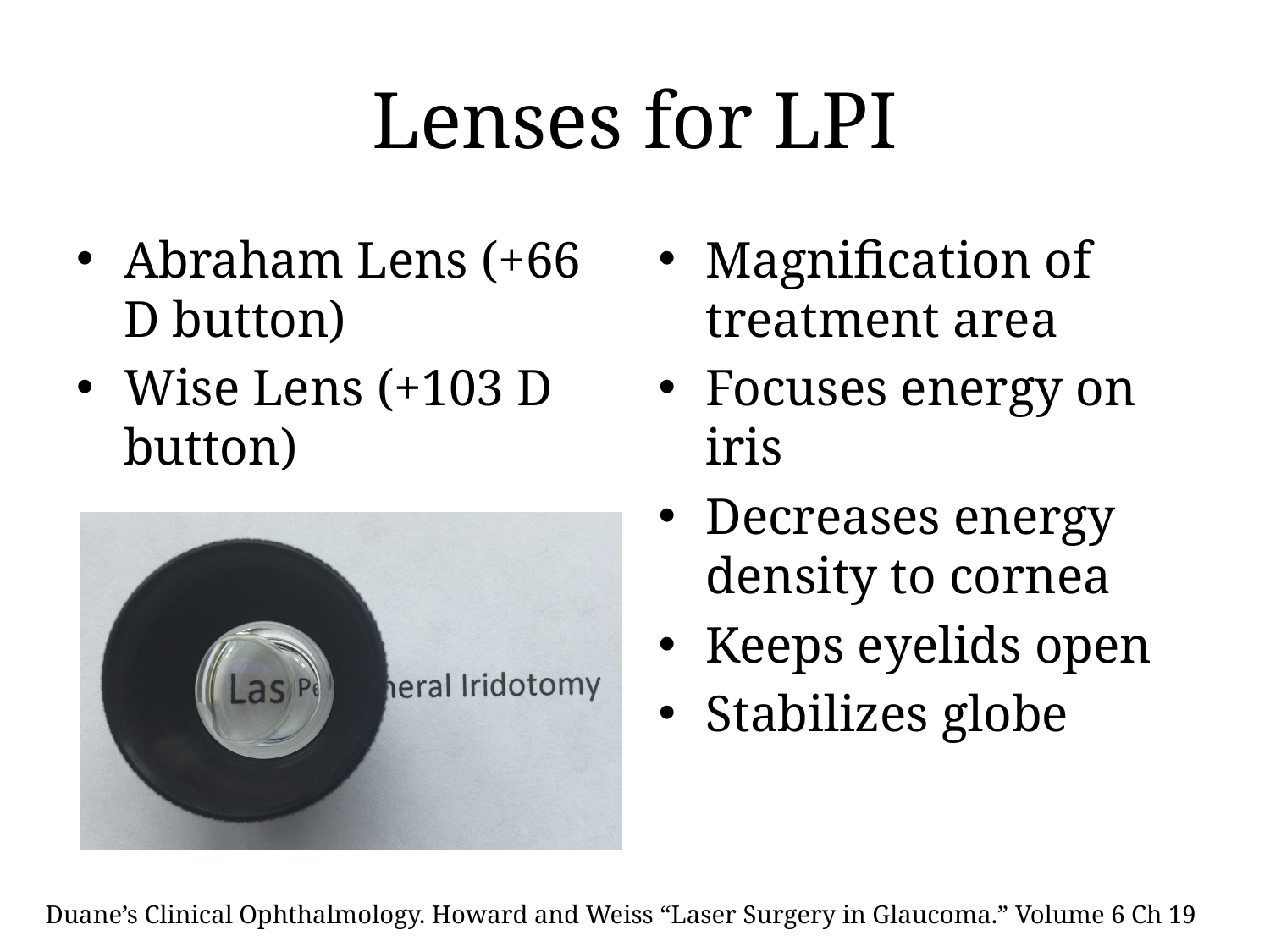

# Lenses for LPI
Abraham Lens (+66 D button)
Wise Lens (+103 D button)
Magnification of treatment area
Focuses energy on iris
Decreases energy density to cornea
Keeps eyelids open
Stabilizes globe
Duane’s Clinical Ophthalmology. Howard and Weiss “Laser Surgery in Glaucoma.” Volume 6 Ch 19

## Slide 6
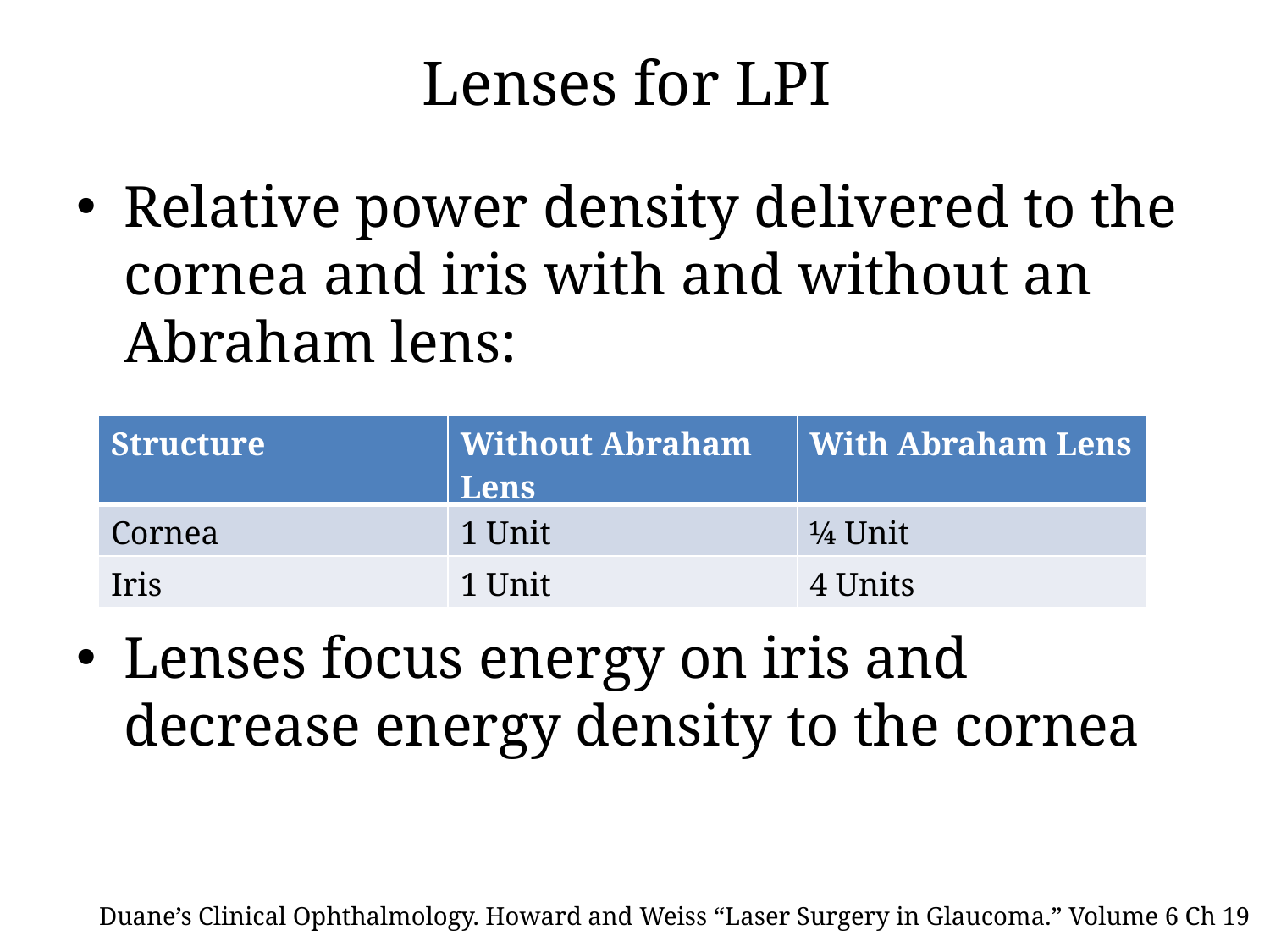

# Lenses for LPI
Relative power density delivered to the cornea and iris with and without an Abraham lens:
Lenses focus energy on iris and decrease energy density to the cornea
| Structure | Without Abraham Lens | With Abraham Lens |
| --- | --- | --- |
| Cornea | 1 Unit | ¼ Unit |
| Iris | 1 Unit | 4 Units |
Duane’s Clinical Ophthalmology. Howard and Weiss “Laser Surgery in Glaucoma.” Volume 6 Ch 19

## Slide 7
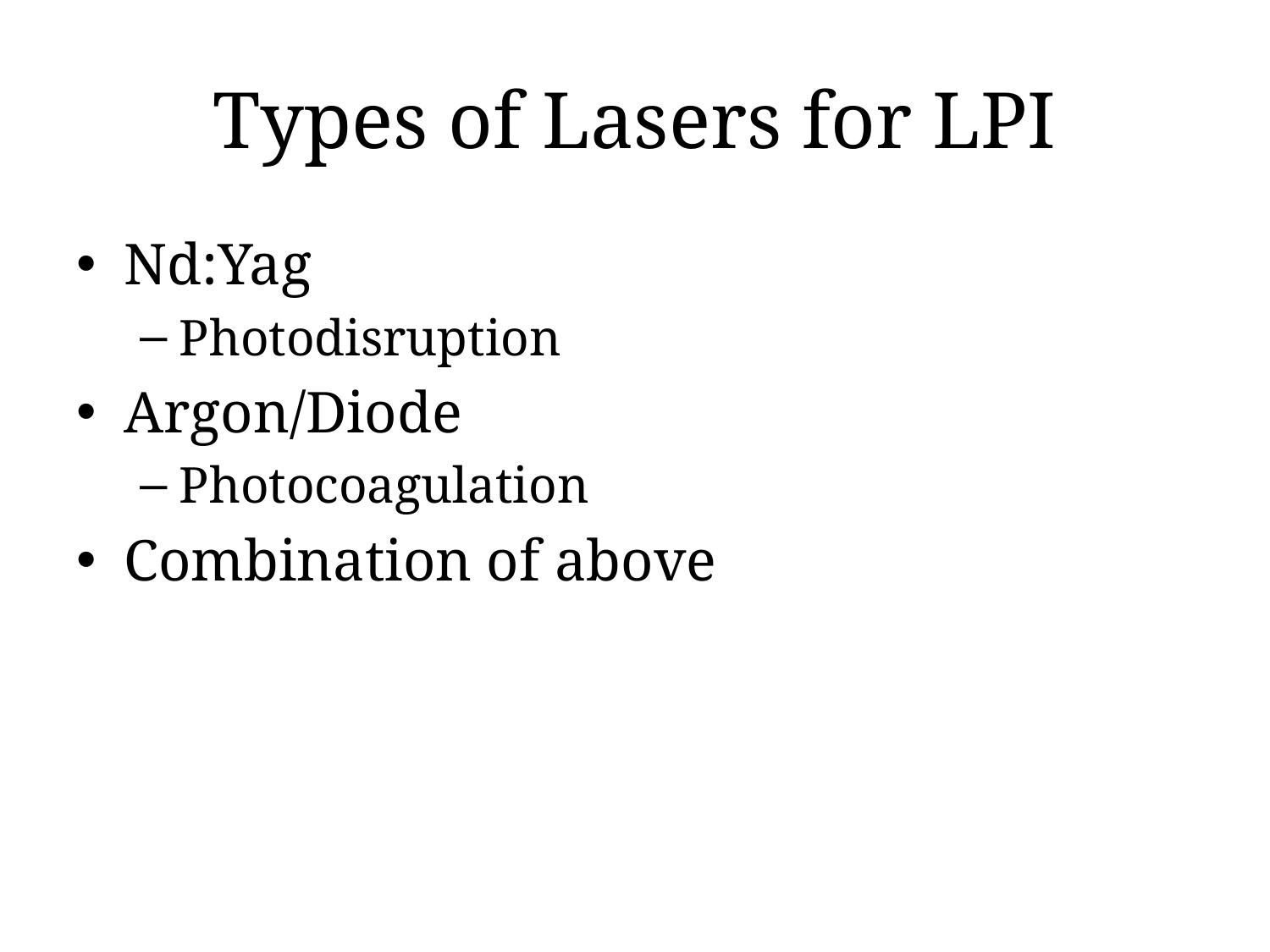

# Types of Lasers for LPI
Nd:Yag
Photodisruption
Argon/Diode
Photocoagulation
Combination of above

## Slide 8
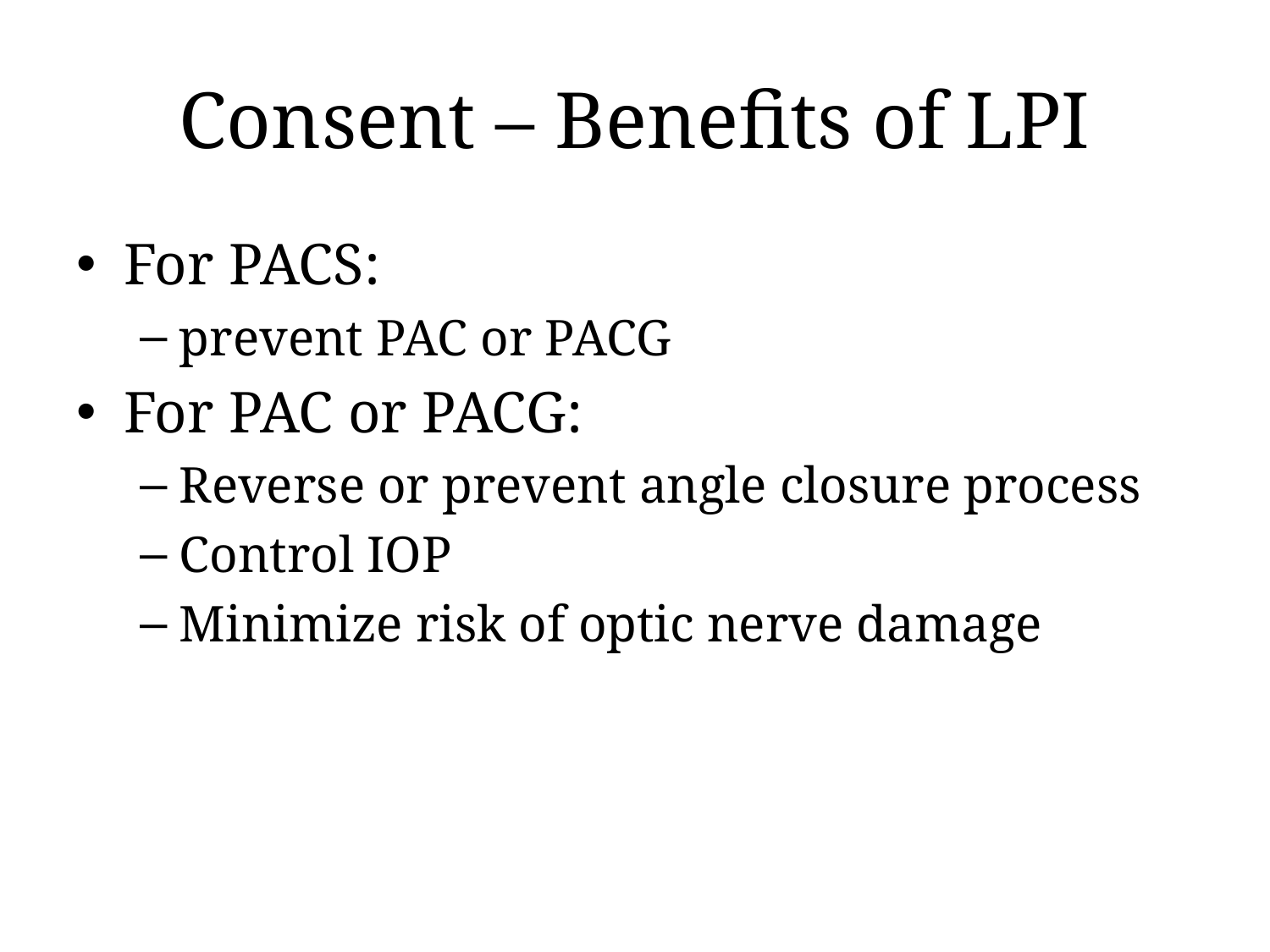

# Consent – Benefits of LPI
For PACS:
prevent PAC or PACG
For PAC or PACG:
Reverse or prevent angle closure process
Control IOP
Minimize risk of optic nerve damage

## Slide 9
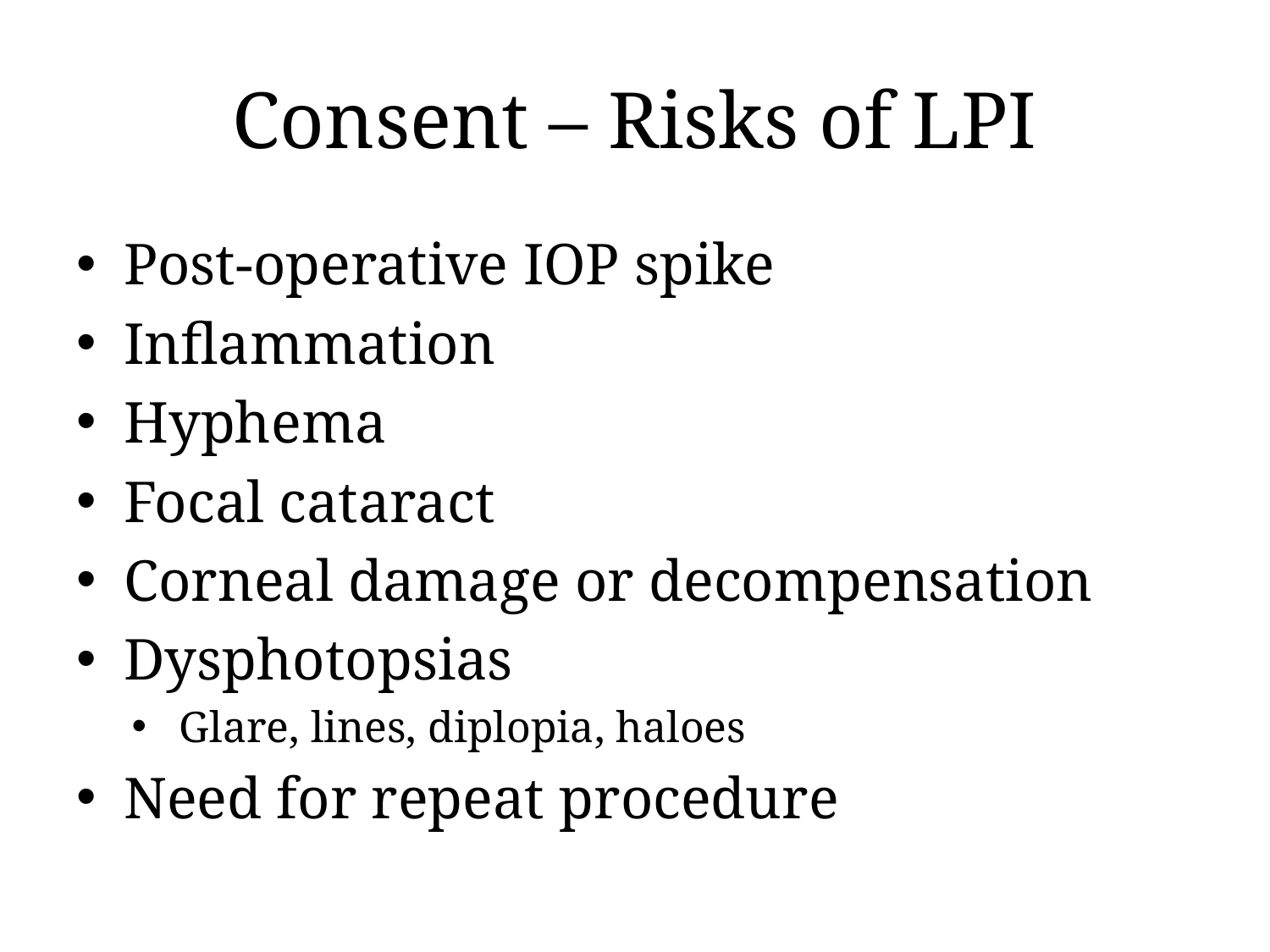

# Consent – Risks of LPI
Post-operative IOP spike
Inflammation
Hyphema
Focal cataract
Corneal damage or decompensation
Dysphotopsias
Glare, lines, diplopia, haloes
Need for repeat procedure

## Slide 10
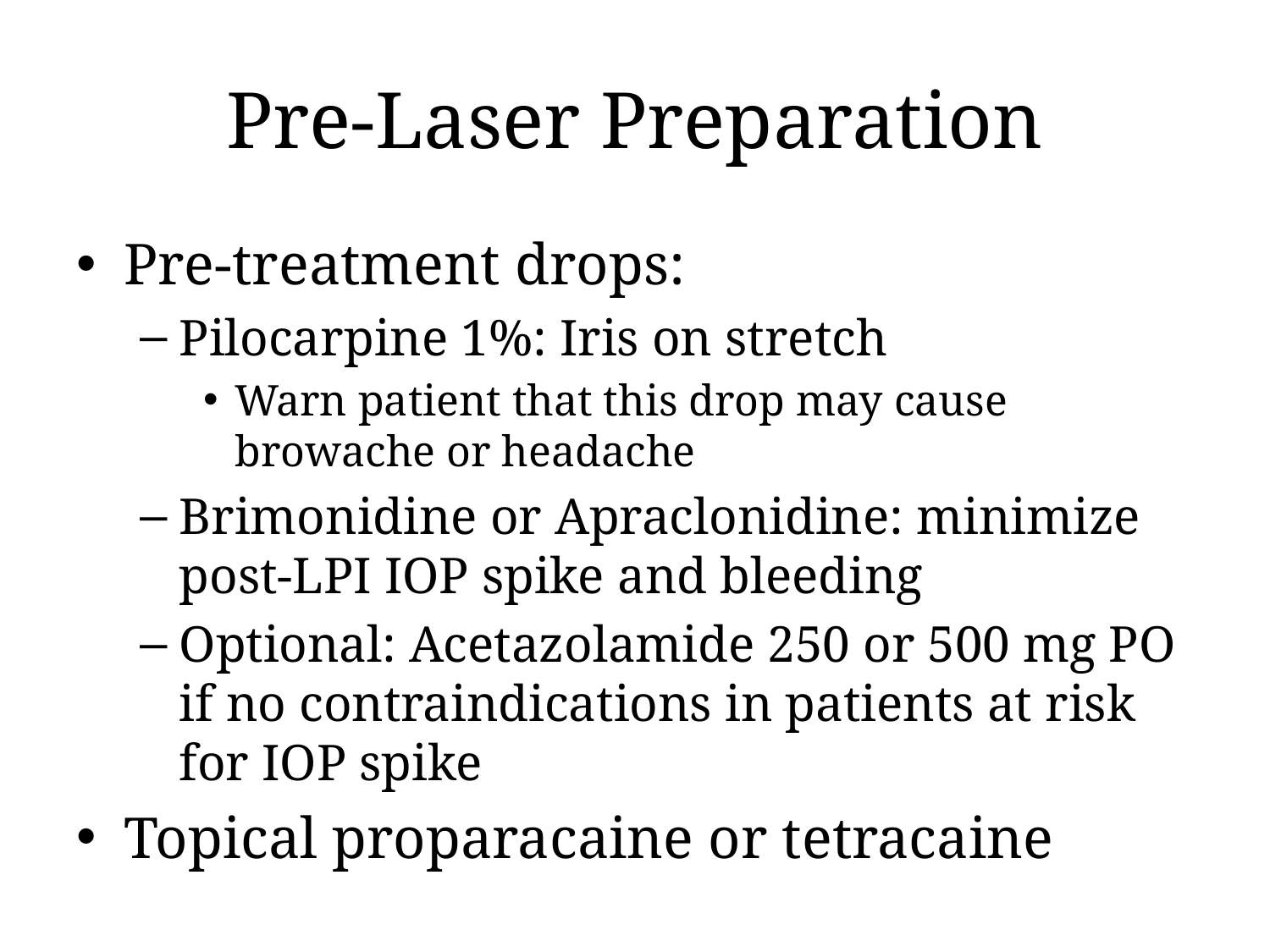

# Pre-Laser Preparation
Pre-treatment drops:
Pilocarpine 1%: Iris on stretch
Warn patient that this drop may cause browache or headache
Brimonidine or Apraclonidine: minimize post-LPI IOP spike and bleeding
Optional: Acetazolamide 250 or 500 mg PO if no contraindications in patients at risk for IOP spike
Topical proparacaine or tetracaine

## Slide 11
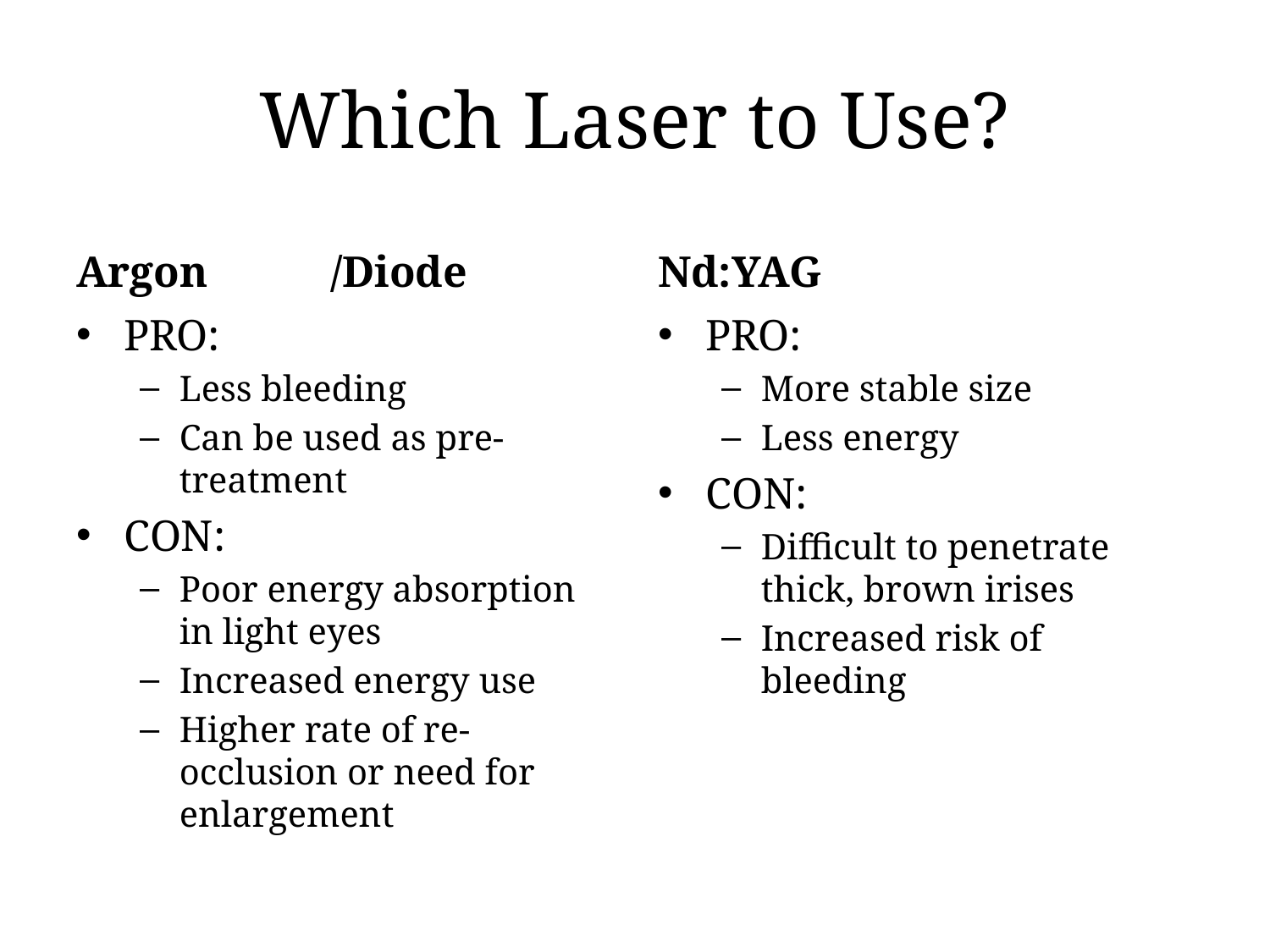

# Which Laser to Use?
Argon	/Diode
Nd:YAG
PRO:
Less bleeding
Can be used as pre-treatment
CON:
Poor energy absorption in light eyes
Increased energy use
Higher rate of re-occlusion or need for enlargement
PRO:
More stable size
Less energy
CON:
Difficult to penetrate thick, brown irises
Increased risk of bleeding

## Slide 12
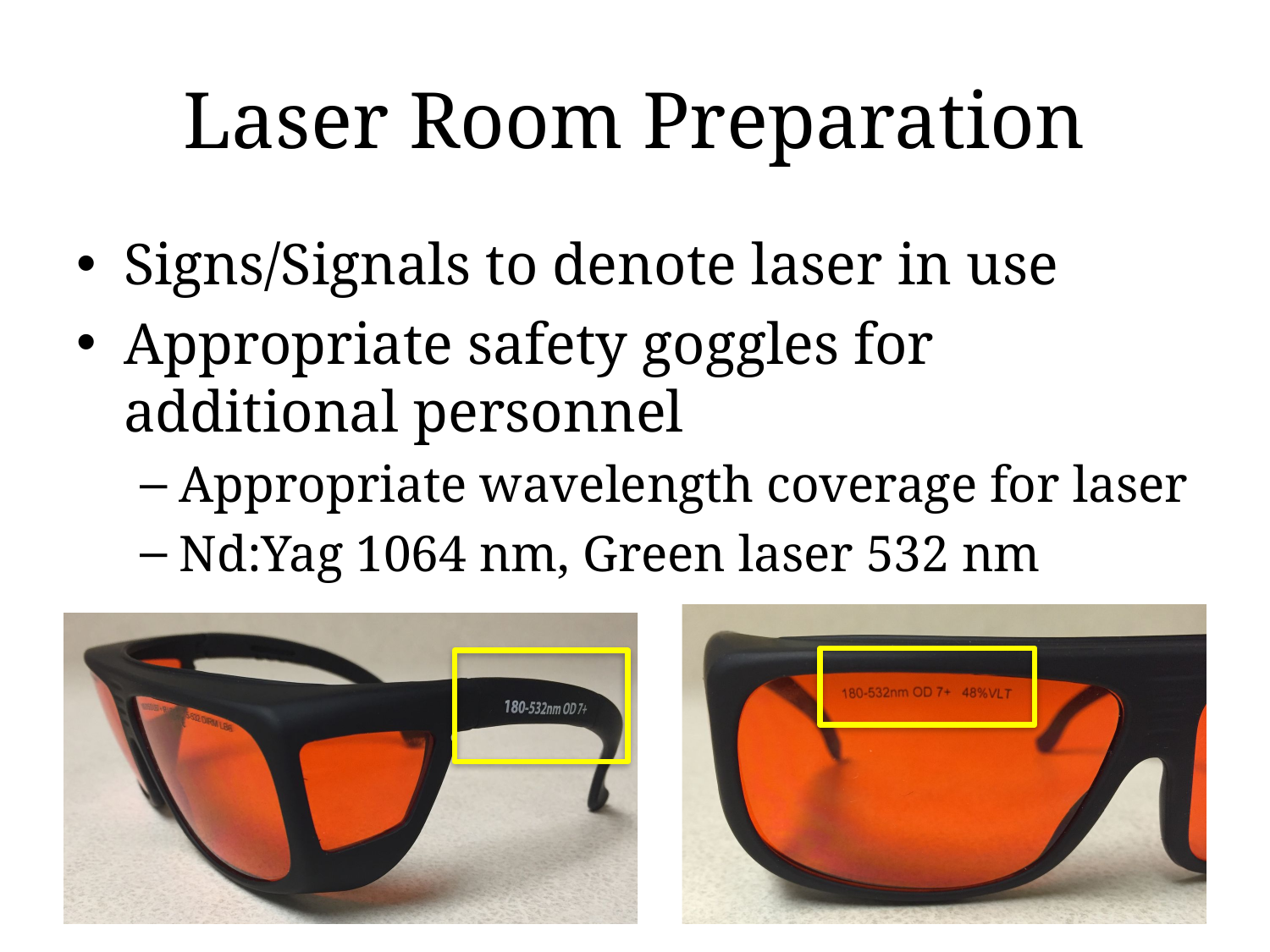

# Laser Room Preparation
Signs/Signals to denote laser in use
Appropriate safety goggles for additional personnel
Appropriate wavelength coverage for laser
Nd:Yag 1064 nm, Green laser 532 nm

## Slide 13
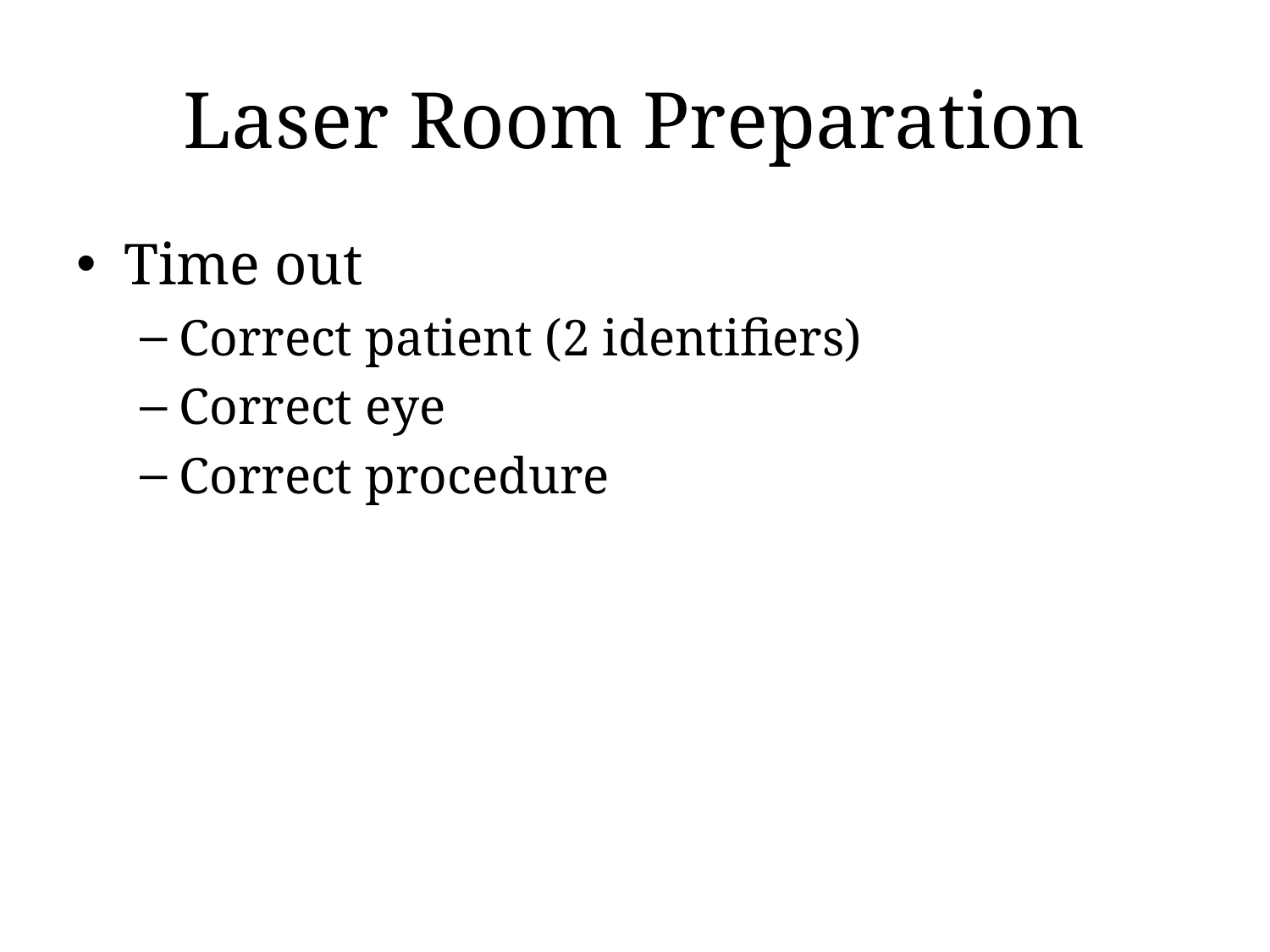

# Laser Room Preparation
Time out
Correct patient (2 identifiers)
Correct eye
Correct procedure

## Slide 14
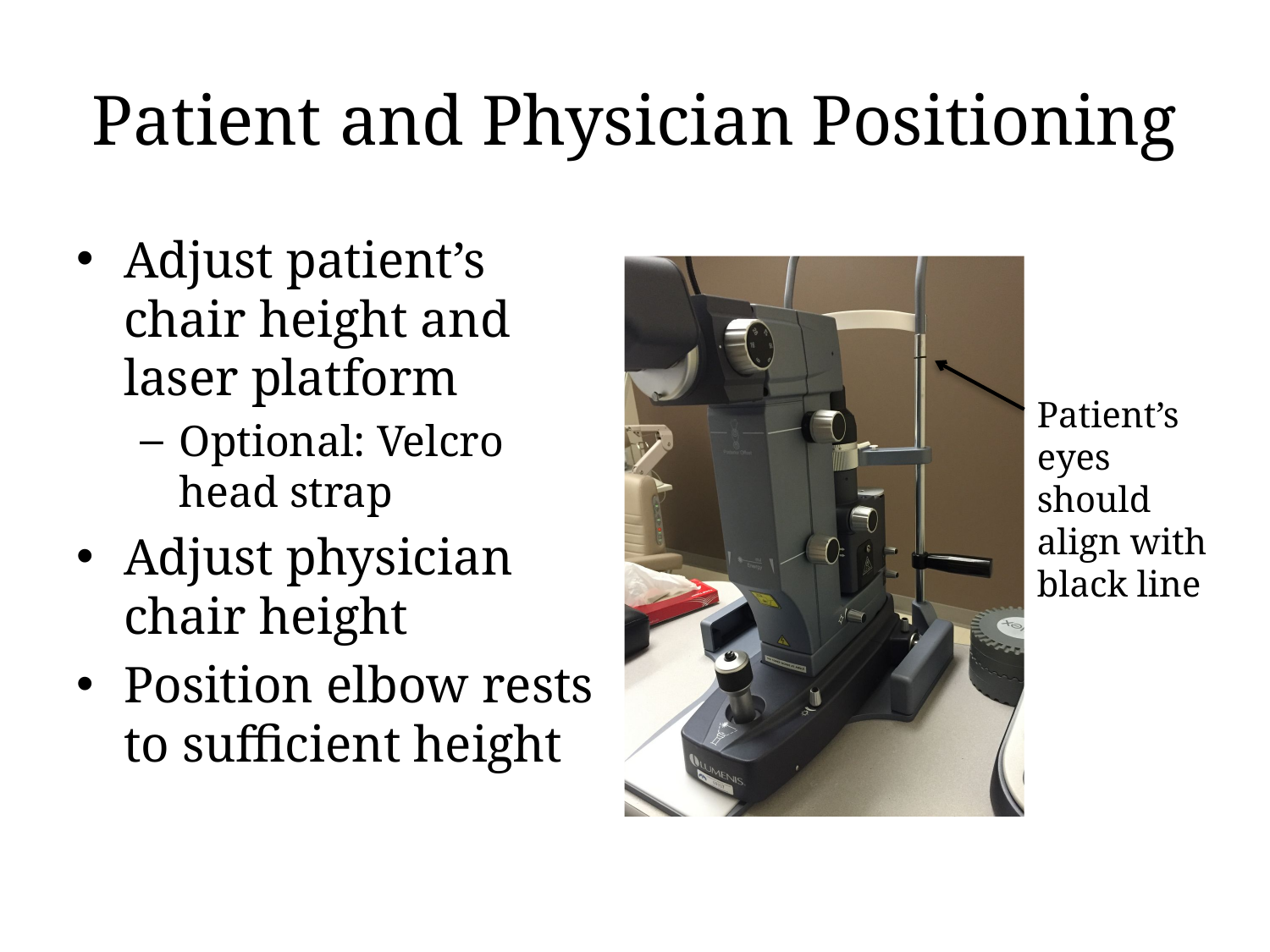

# Patient and Physician Positioning
Adjust patient’s chair height and laser platform
Optional: Velcro head strap
Adjust physician chair height
Position elbow rests to sufficient height
Patient’s eyes should align with black line

## Slide 15
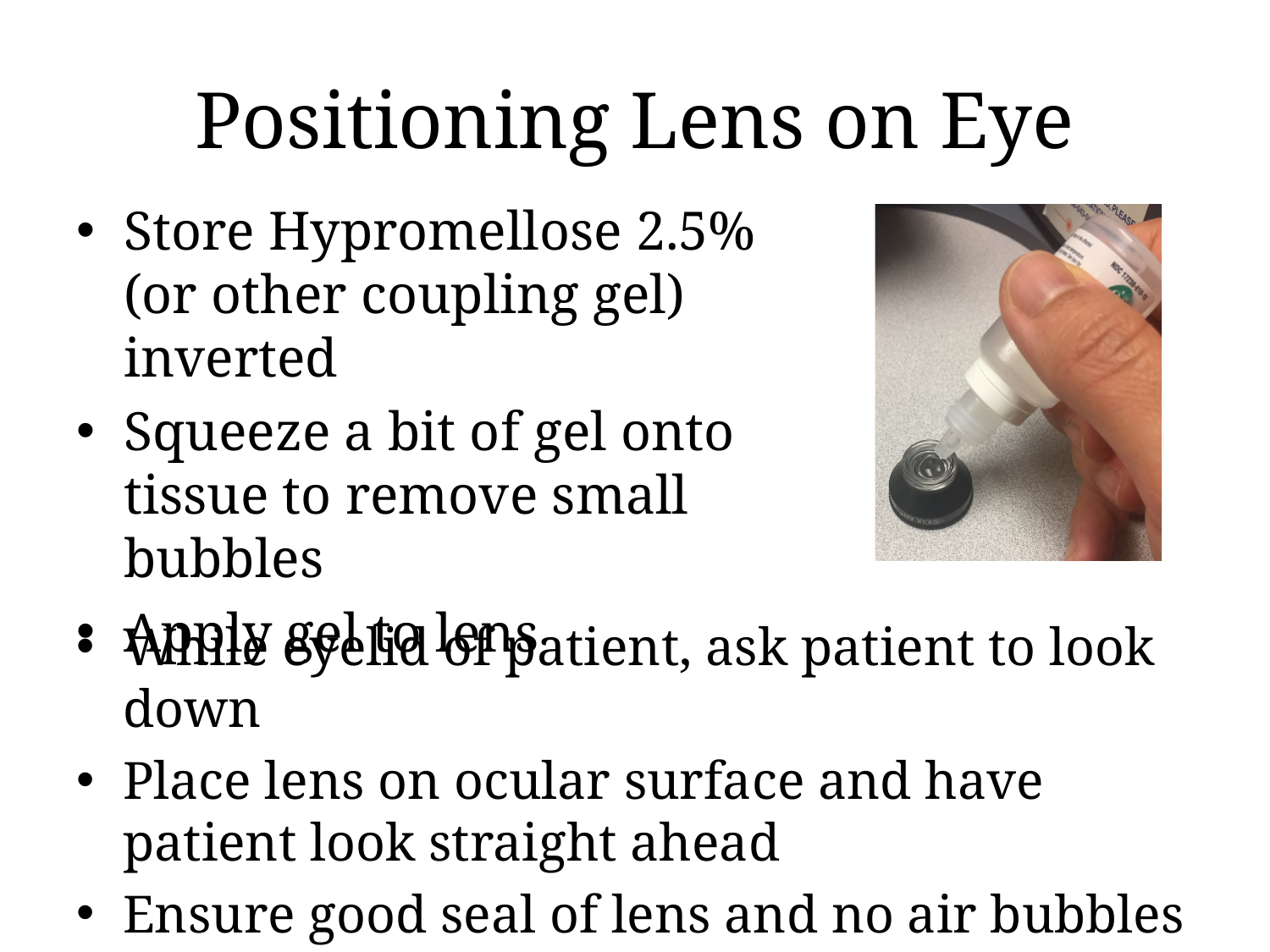

# Positioning Lens on Eye
Store Hypromellose 2.5% (or other coupling gel) inverted
Squeeze a bit of gel onto tissue to remove small bubbles
Apply gel to lens
While eyelid of patient, ask patient to look down
Place lens on ocular surface and have patient look straight ahead
Ensure good seal of lens and no air bubbles

## Slide 16
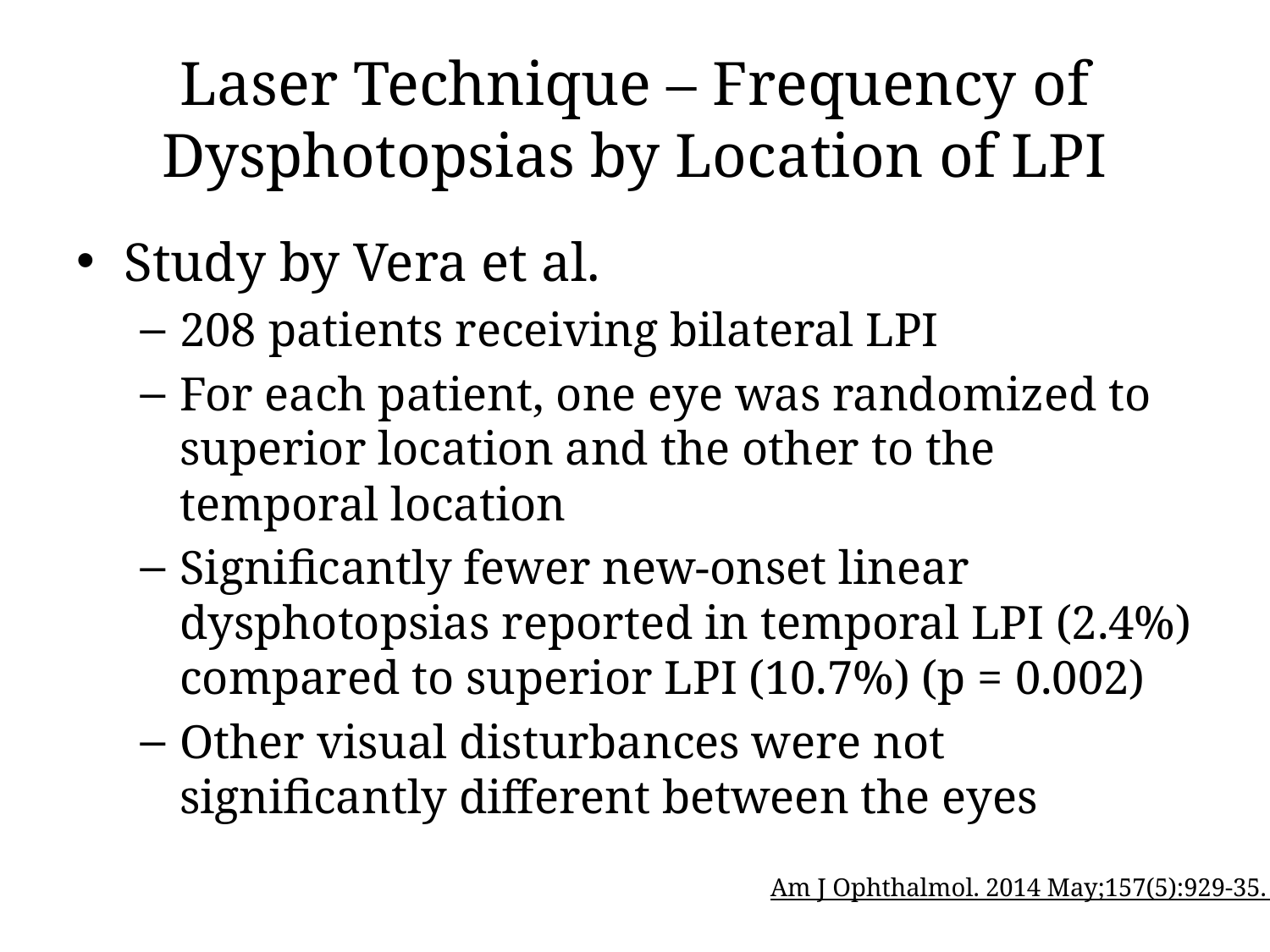

# Laser Technique – Frequency of Dysphotopsias by Location of LPI
Study by Vera et al.
208 patients receiving bilateral LPI
For each patient, one eye was randomized to superior location and the other to the temporal location
Significantly fewer new-onset linear dysphotopsias reported in temporal LPI (2.4%) compared to superior LPI (10.7%) (p = 0.002)
Other visual disturbances were not significantly different between the eyes
Am J Ophthalmol. 2014 May;157(5):929-35.

## Slide 17
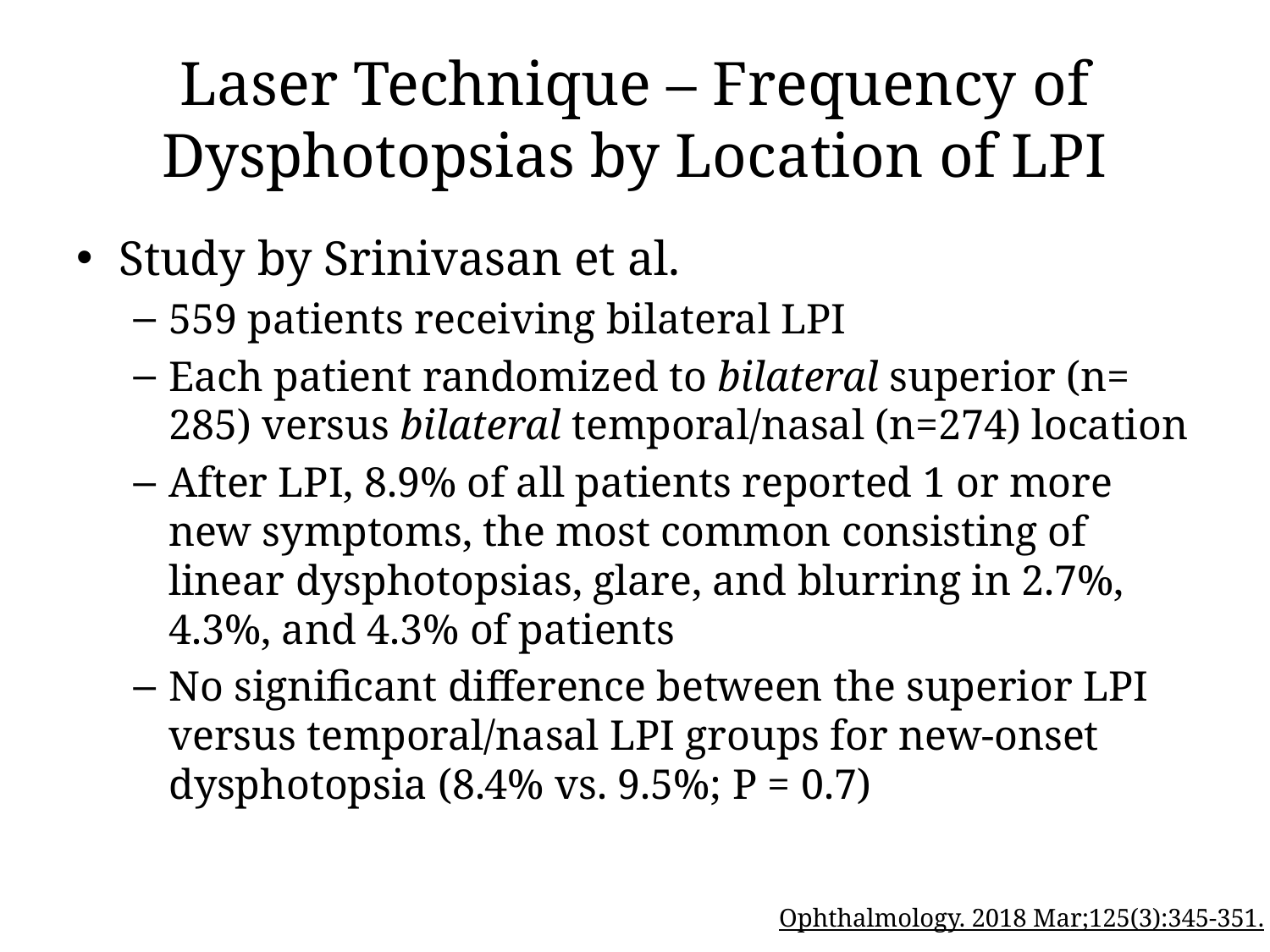

# Laser Technique – Frequency of Dysphotopsias by Location of LPI
Study by Srinivasan et al.
559 patients receiving bilateral LPI
Each patient randomized to bilateral superior (n= 285) versus bilateral temporal/nasal (n=274) location
After LPI, 8.9% of all patients reported 1 or more new symptoms, the most common consisting of linear dysphotopsias, glare, and blurring in 2.7%, 4.3%, and 4.3% of patients
No significant difference between the superior LPI versus temporal/nasal LPI groups for new-onset dysphotopsia (8.4% vs. 9.5%; P = 0.7)
Ophthalmology. 2018 Mar;125(3):345-351.

## Slide 18
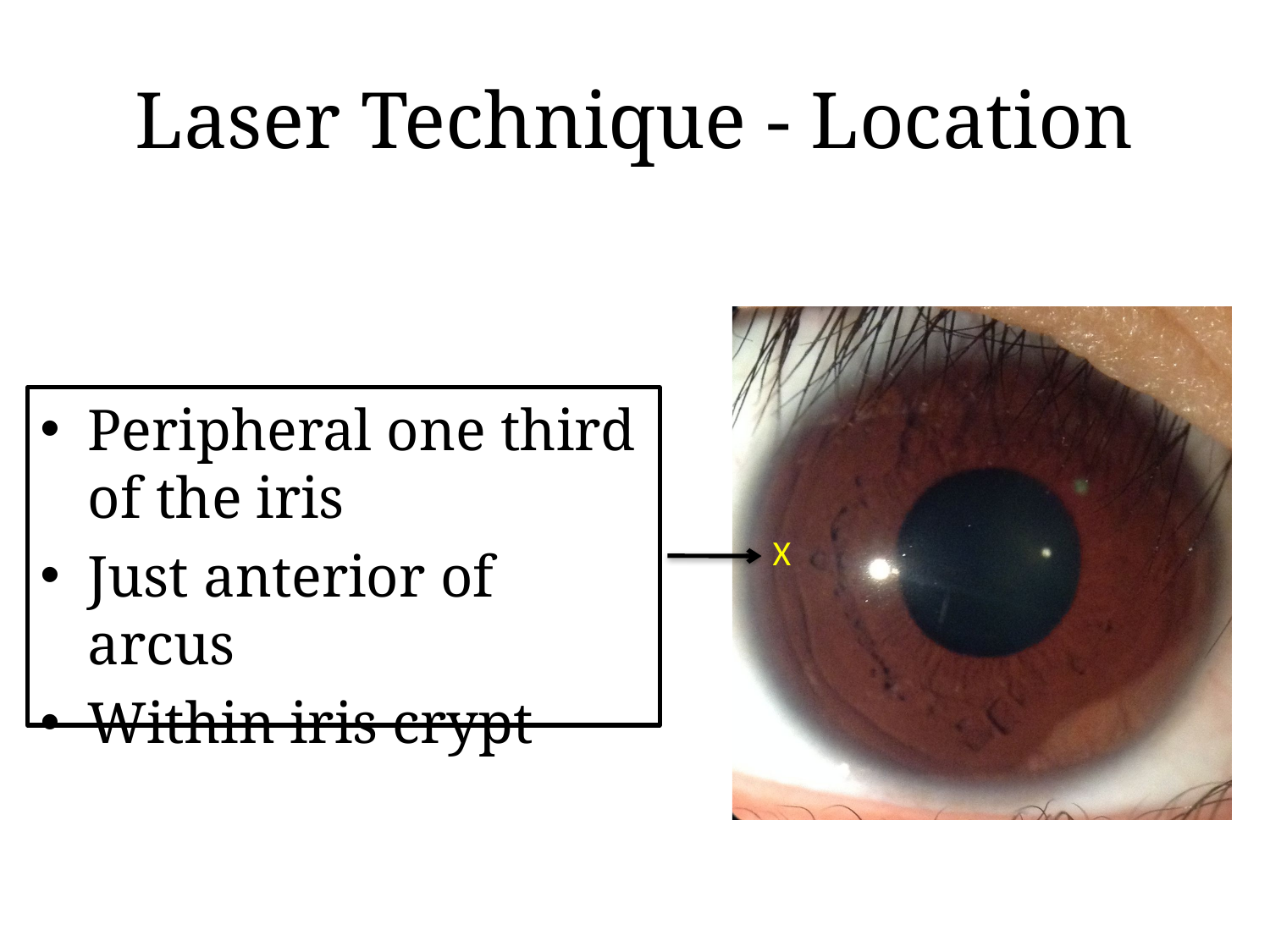

# Laser Technique - Location
Peripheral one third of the iris
Just anterior of arcus
Within iris crypt
X

## Slide 19
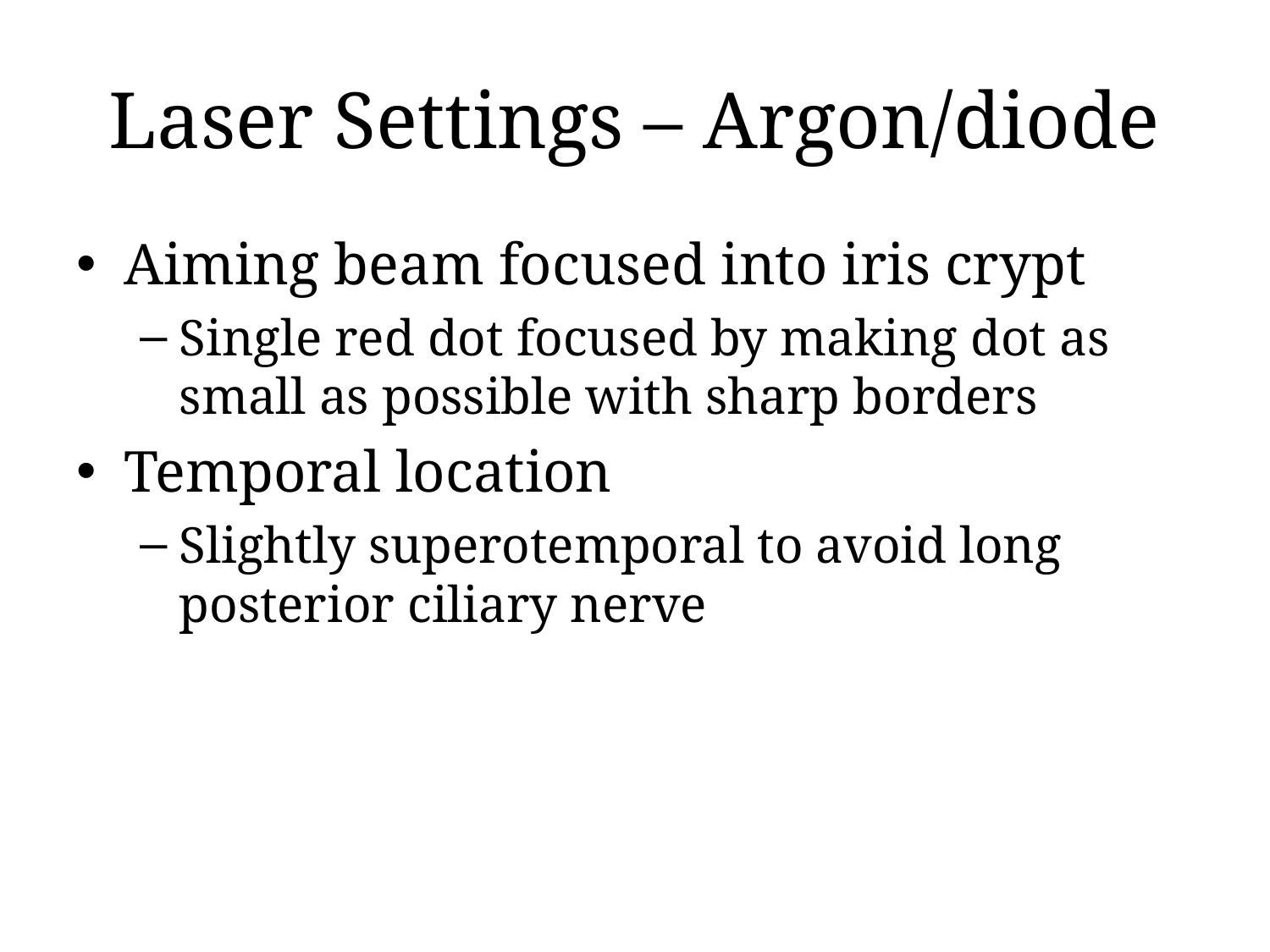

# Laser Settings – Argon/diode
Aiming beam focused into iris crypt
Single red dot focused by making dot as small as possible with sharp borders
Temporal location
Slightly superotemporal to avoid long posterior ciliary nerve

## Slide 20
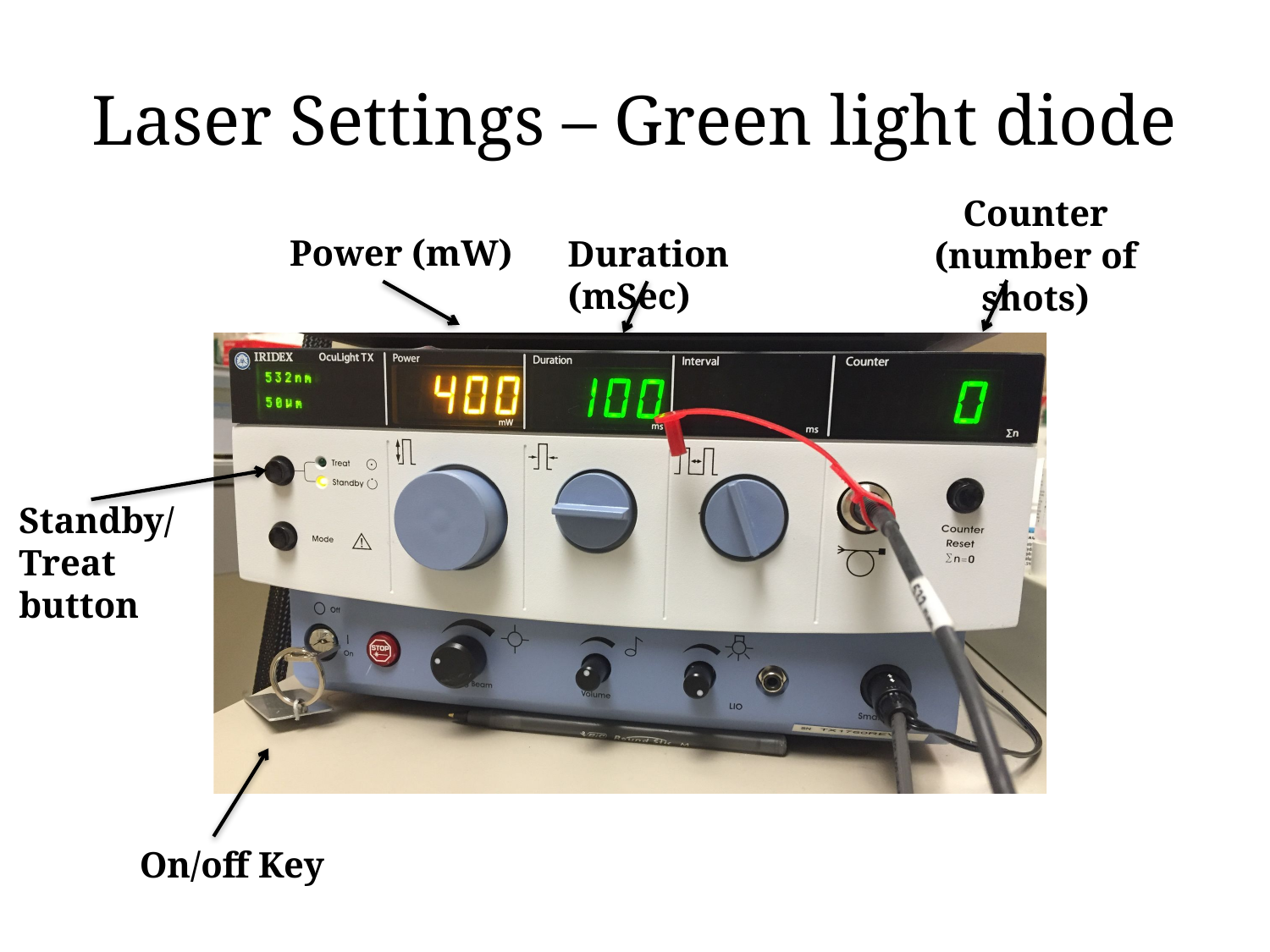

# Laser Settings – Green light diode
Counter
(number of shots)
Power (mW)
Duration (mSec)
Standby/Treat button
On/off Key

## Slide 21
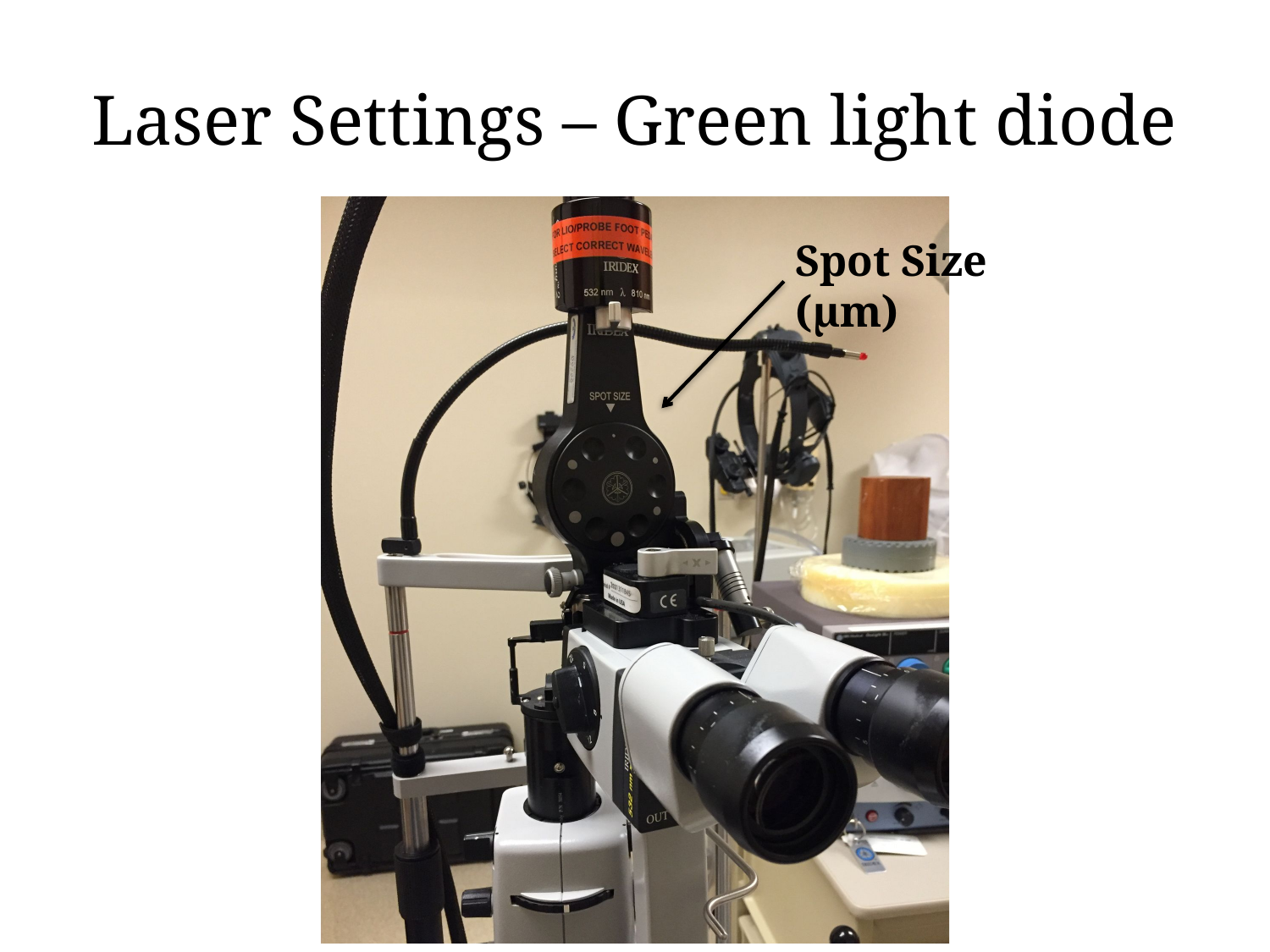

# Laser Settings – Green light diode
Spot Size (µm)

## Slide 22
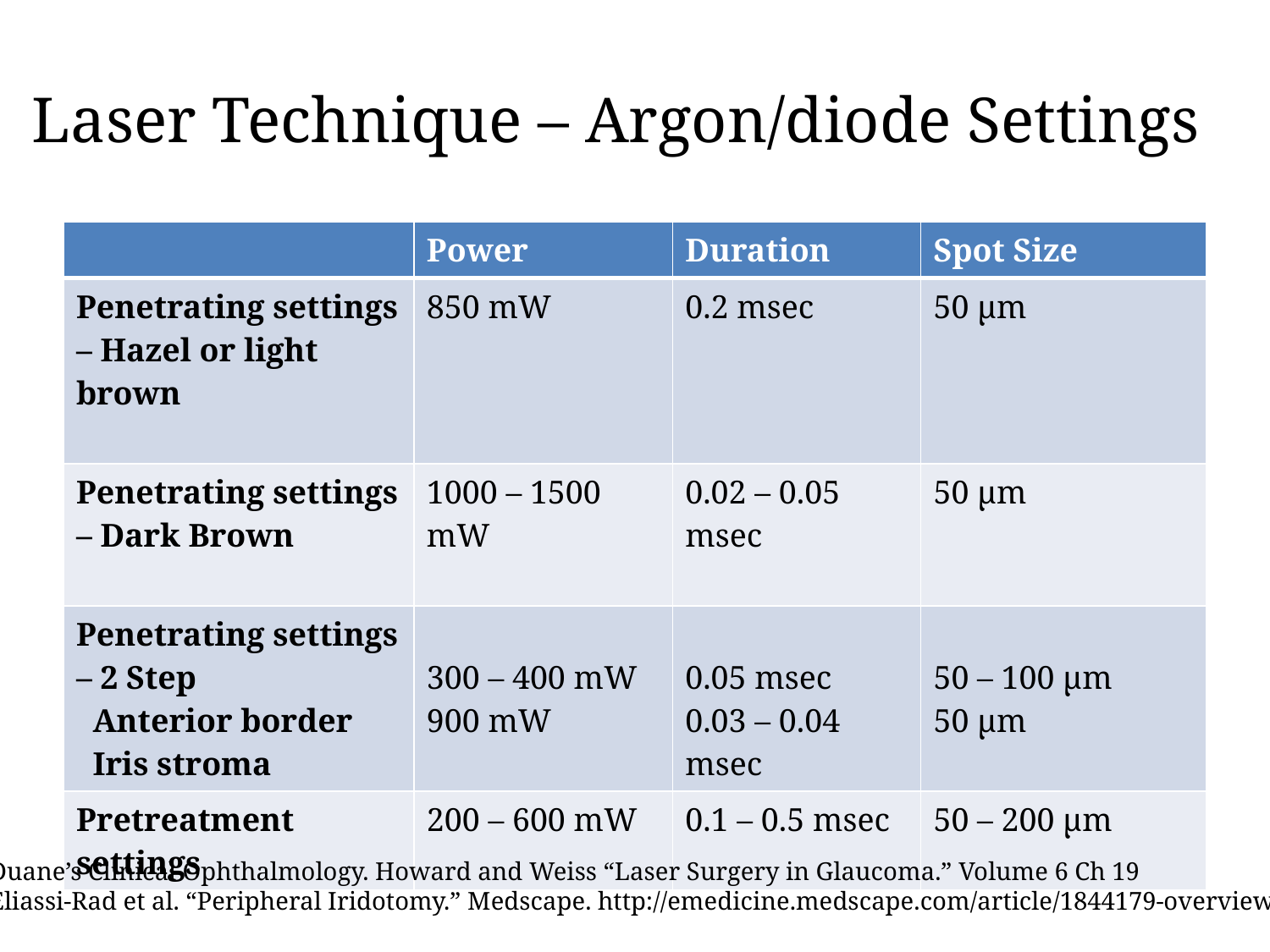

# Laser Technique – Argon/diode Settings
| | Power | Duration | Spot Size |
| --- | --- | --- | --- |
| Penetrating settings – Hazel or light brown | 850 mW | 0.2 msec | 50 µm |
| Penetrating settings – Dark Brown | 1000 – 1500 mW | 0.02 – 0.05 msec | 50 µm |
| Penetrating settings – 2 Step Anterior border Iris stroma | 300 – 400 mW 900 mW | 0.05 msec 0.03 – 0.04 msec | 50 – 100 µm 50 µm |
| Pretreatment settings | 200 – 600 mW | 0.1 – 0.5 msec | 50 – 200 µm |
Duane’s Clinical Ophthalmology. Howard and Weiss “Laser Surgery in Glaucoma.” Volume 6 Ch 19
Eliassi-Rad et al. “Peripheral Iridotomy.” Medscape. http://emedicine.medscape.com/article/1844179-overview

## Slide 23
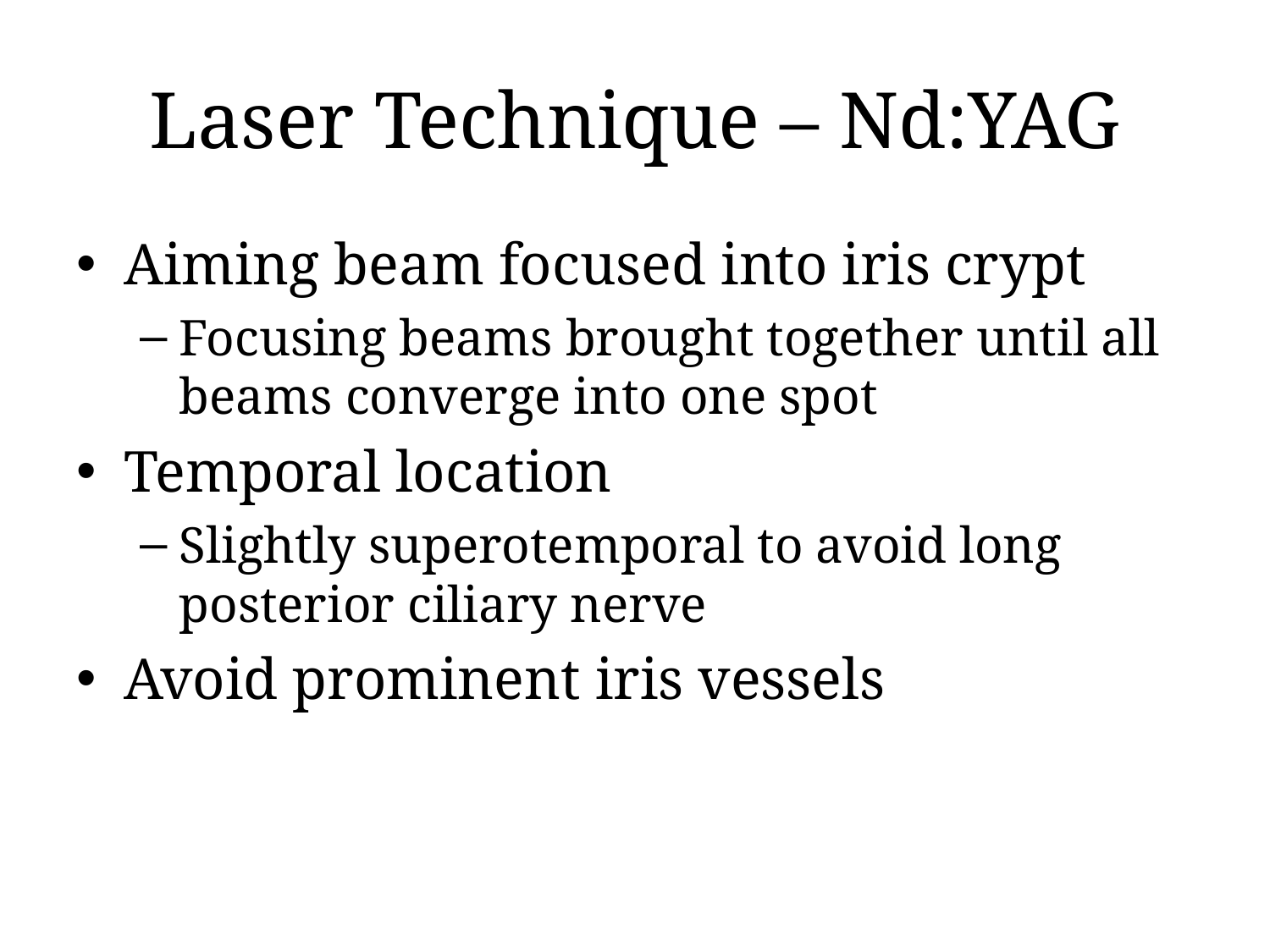

# Laser Technique – Nd:YAG
Aiming beam focused into iris crypt
Focusing beams brought together until all beams converge into one spot
Temporal location
Slightly superotemporal to avoid long posterior ciliary nerve
Avoid prominent iris vessels

## Slide 24
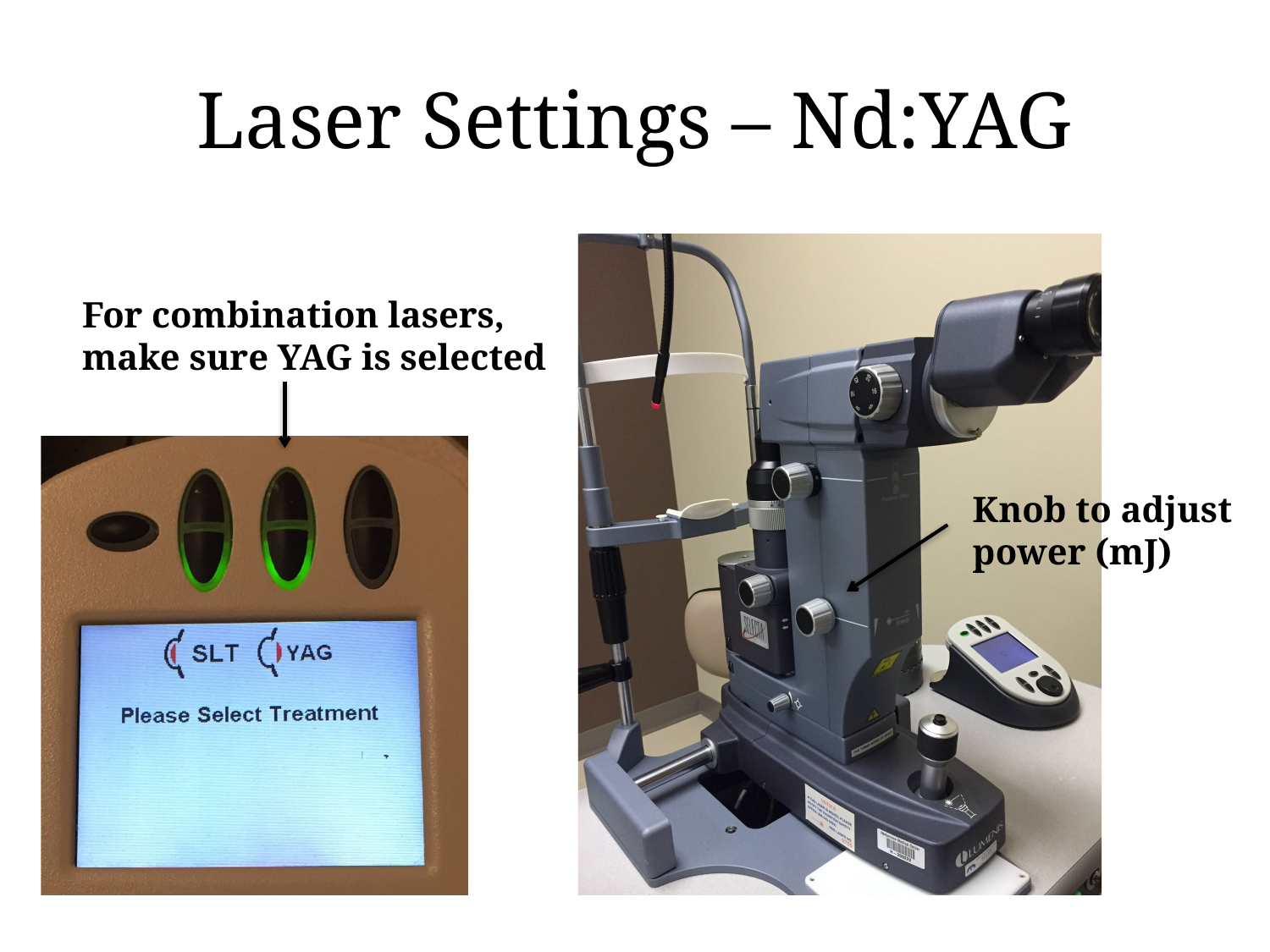

# Laser Settings – Nd:YAG
For combination lasers, make sure YAG is selected
Knob to adjust power (mJ)

## Slide 25
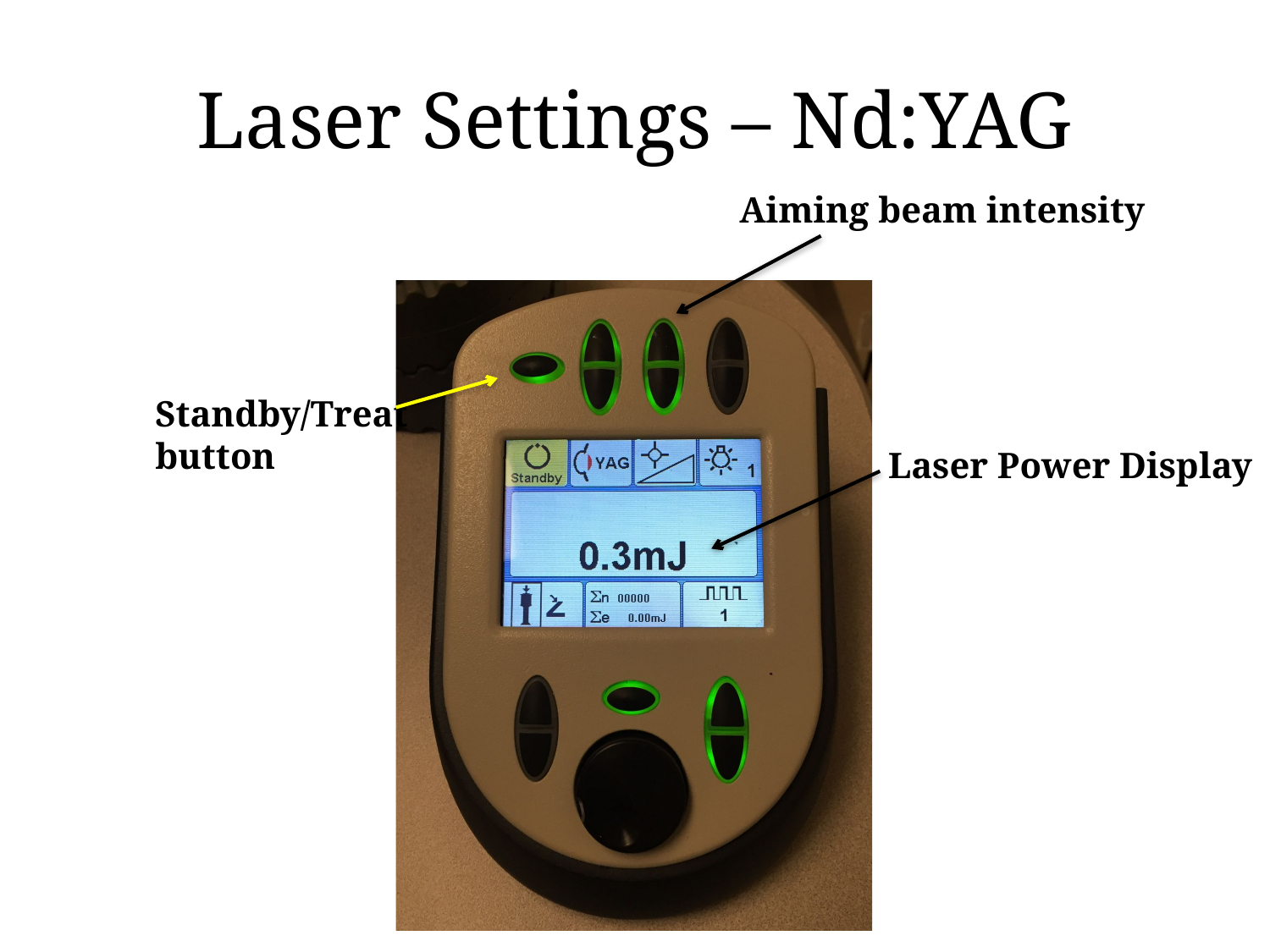

# Laser Settings – Nd:YAG
Aiming beam intensity
Standby/Treat button
Laser Power Display

## Slide 26
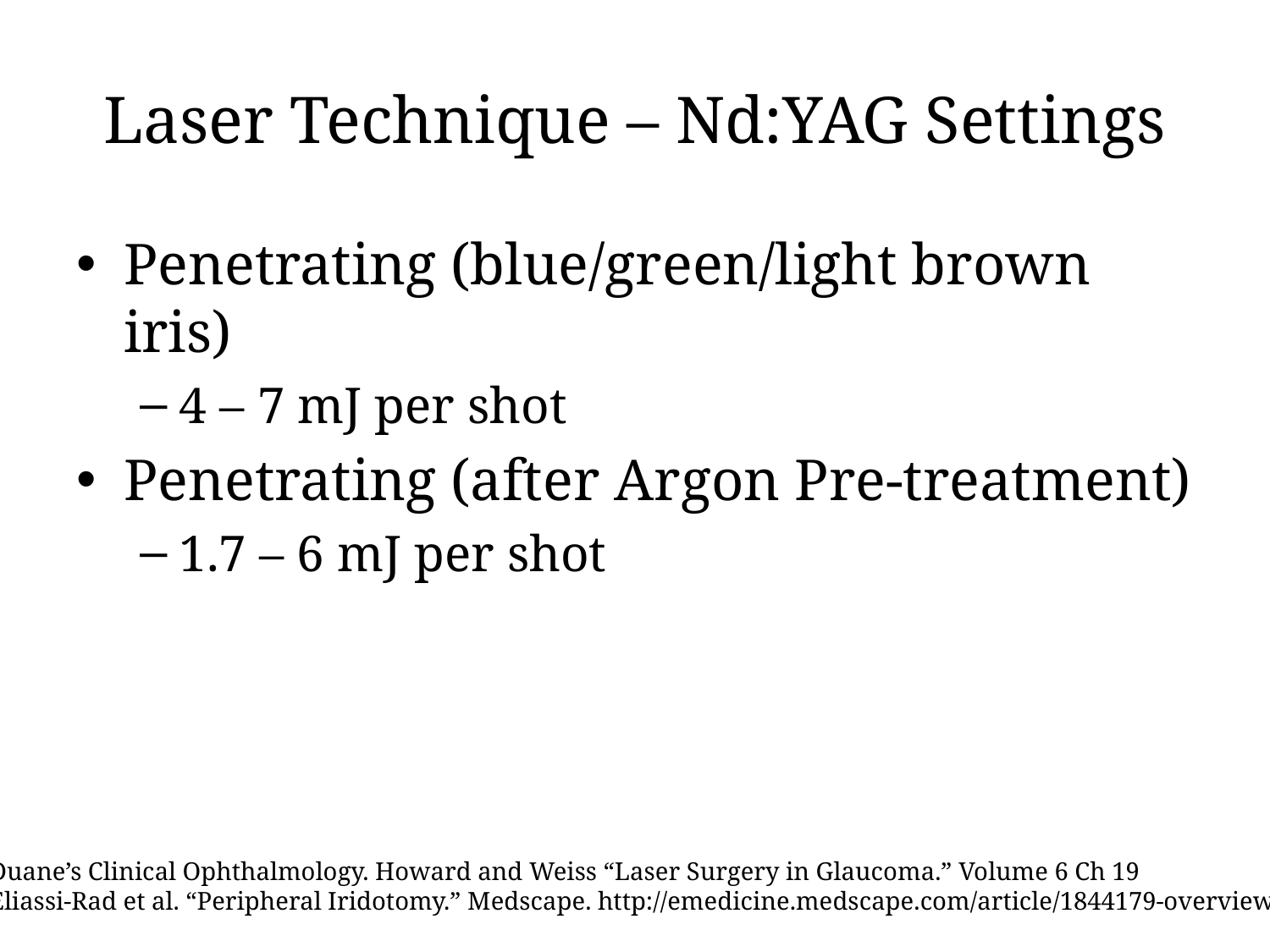

# Laser Technique – Nd:YAG Settings
Penetrating (blue/green/light brown iris)
4 – 7 mJ per shot
Penetrating (after Argon Pre-treatment)
1.7 – 6 mJ per shot
Duane’s Clinical Ophthalmology. Howard and Weiss “Laser Surgery in Glaucoma.” Volume 6 Ch 19
Eliassi-Rad et al. “Peripheral Iridotomy.” Medscape. http://emedicine.medscape.com/article/1844179-overview

## Slide 27
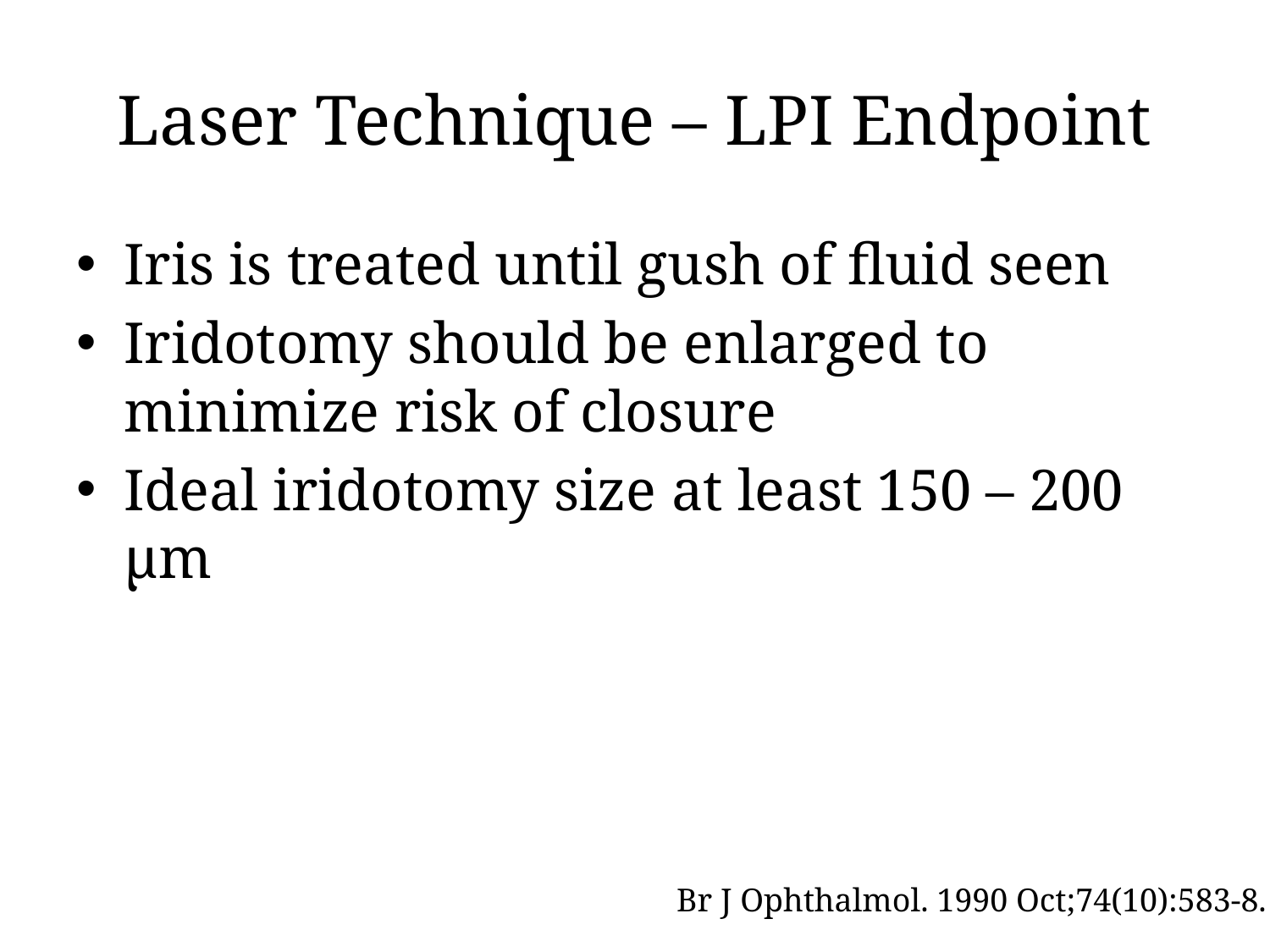

# Laser Technique – LPI Endpoint
Iris is treated until gush of fluid seen
Iridotomy should be enlarged to minimize risk of closure
Ideal iridotomy size at least 150 – 200 µm
Br J Ophthalmol. 1990 Oct;74(10):583-8.

## Slide 28
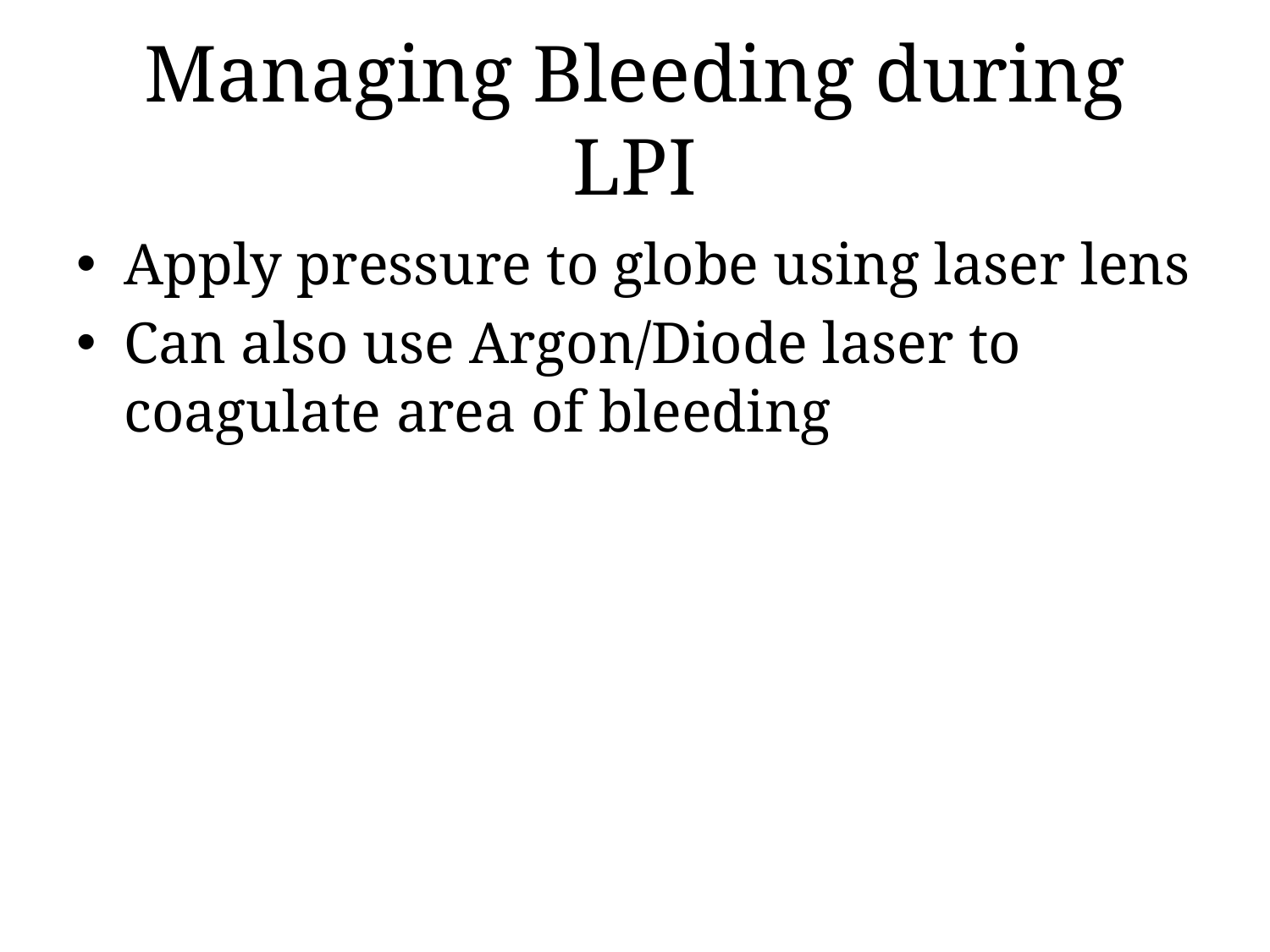

# Managing Bleeding during LPI
Apply pressure to globe using laser lens
Can also use Argon/Diode laser to coagulate area of bleeding

## Slide 29
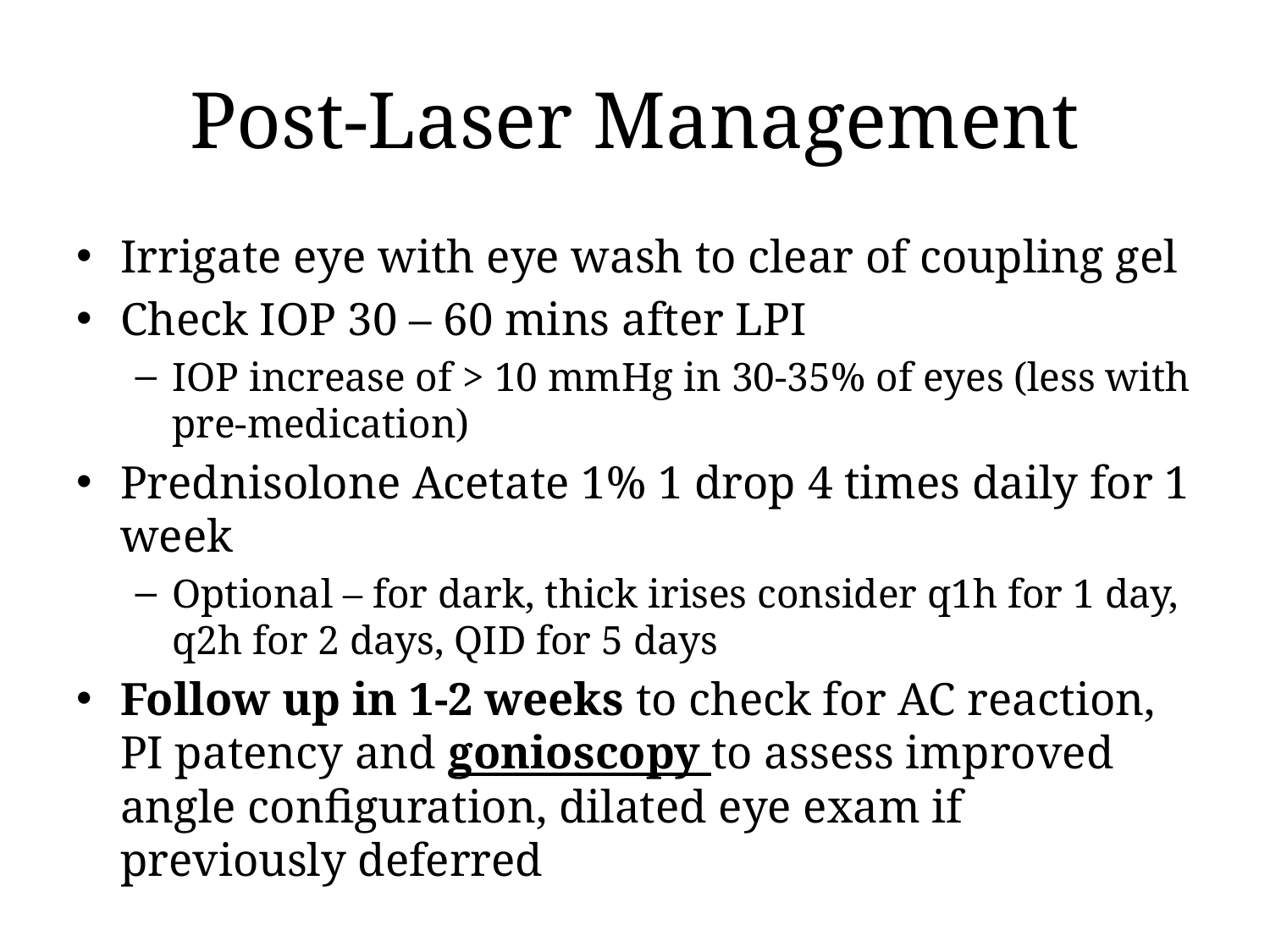

# Post-Laser Management
Irrigate eye with eye wash to clear of coupling gel
Check IOP 30 – 60 mins after LPI
IOP increase of > 10 mmHg in 30-35% of eyes (less with pre-medication)
Prednisolone Acetate 1% 1 drop 4 times daily for 1 week
Optional – for dark, thick irises consider q1h for 1 day, q2h for 2 days, QID for 5 days
Follow up in 1-2 weeks to check for AC reaction, PI patency and gonioscopy to assess improved angle configuration, dilated eye exam if previously deferred

## Slide 30
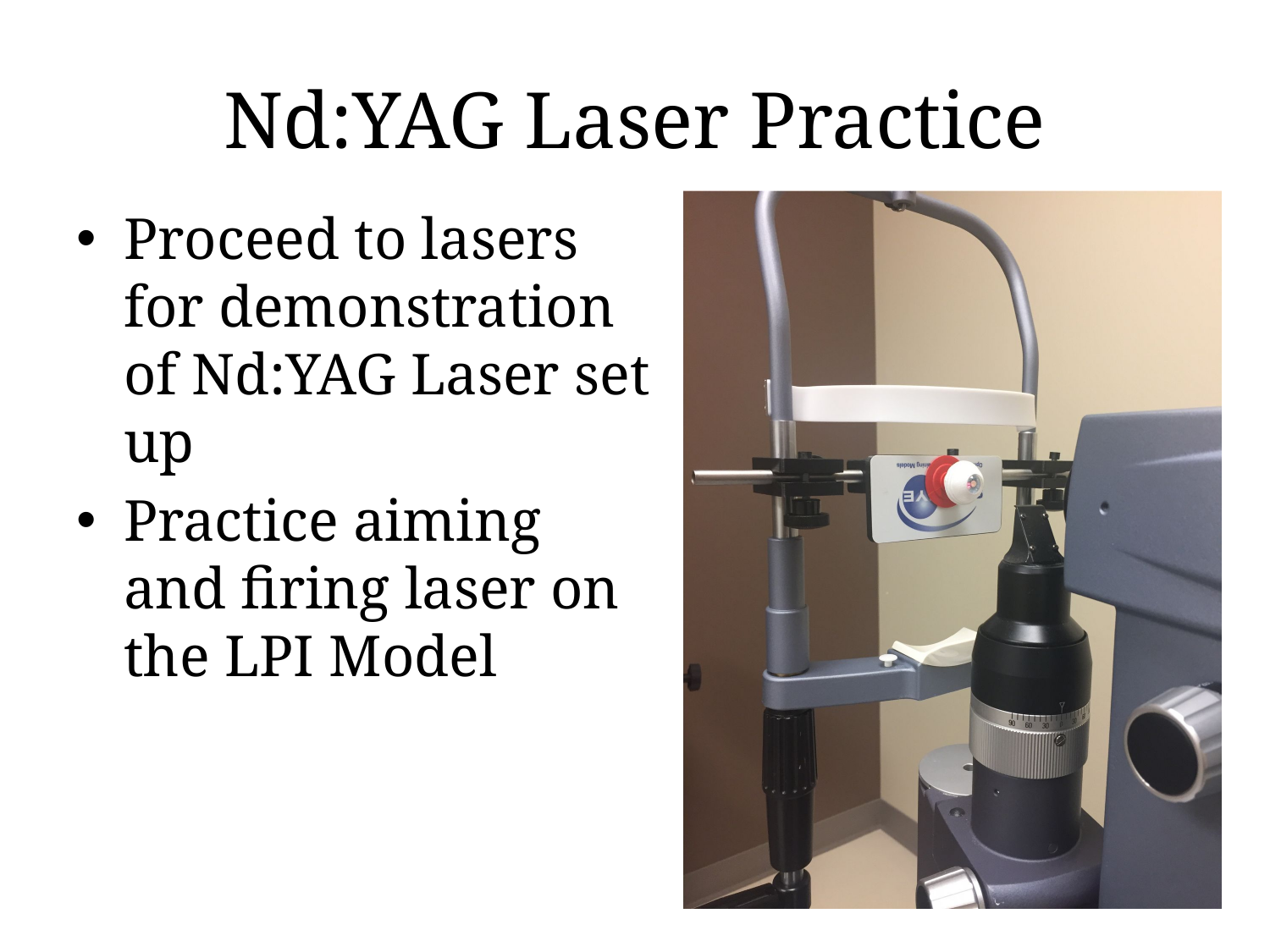

# Nd:YAG Laser Practice
Proceed to lasers for demonstration of Nd:YAG Laser set up
Practice aiming and firing laser on the LPI Model
