# Supplementary material for: Laser Peripheral Iridotomy Curriculum: Lecture and Simulation Practical
Source: MedEdPORTAL. 2020 May 27;16:10903. doi: 10.15766/mep_2374-8265.10903 (PMC7331967; doi:10.15766/mep_2374-8265.10903)
Supplement: Supplementary file 1 — Pretest.docxLecture and Notes.pptxInitial LPI Assessment.docxFinal LPI Assessment.docxPosttest.docxPre- & Posttest Answers.docx [file mep_2374-8265.10903-s001.zip › C. Initial LPI Assessment.docx]

**Resident Name:_________________________________________________**

Total number of spots to complete LPI: __________________

Time from lens placed on model to complete LPI:_________________

**Resident Name:_________________________________________________**

Total number of spots to complete LPI: __________________

Time from lens placed on model to complete LPI:_________________

**Resident Name:_________________________________________________**

Total number of spots to complete LPI: __________________

Time from lens placed on model to complete LPI:_________________

**Resident Name:_________________________________________________**

Total number of spots to complete LPI: __________________

Time from lens placed on model to complete LPI:_________________

**Resident Name:_________________________________________________**

Total number of spots to complete LPI: __________________

Time from lens placed on model to complete LPI:_________________
