# Supplementary material for: Laser Peripheral Iridotomy Curriculum: Lecture and Simulation Practical
Source: MedEdPORTAL. 2020 May 27;16:10903. doi: 10.15766/mep_2374-8265.10903 (PMC7331967; doi:10.15766/mep_2374-8265.10903)
Supplement: Supplementary file 1 — Pretest.docxLecture and Notes.pptxInitial LPI Assessment.docxFinal LPI Assessment.docxPosttest.docxPre- & Posttest Answers.docx [file mep_2374-8265.10903-s001.zip › D. Final LPI Assessment.docx]

**Assessment of Proper Use of Laser Equipment:**

[] Select proper eye protection (must pick appropriate wavelength coverage)

[] Turn laser on

[] Select correct laser setting (YAG)

[] Demonstrate how to change aiming beam brightness

[] Demonstrate how to change slit beam brightness

[] Demonstrate how to change slit beam width

[] Demonstrate how to change laser focus (anterior versus posterior)

[] Demonstrate how to change laser power

**Assessment of LPI technique:**

[] Select appropriate initial laser settings

[] Switch from “standby” to “on”

[] Demonstrate placement of Goniosol on lens

[] Demonstrate proper alignment of slit beam (slit beam must be off axis for

YAG)

[] Demonstrate proper alignment of laser beam to targeted area

Total number of spots to complete LPI: __________________

Total time from gonio lens placed on model to complete LPI:_________________
