# Supplementary material for: Laser Peripheral Iridotomy Curriculum: Lecture and Simulation Practical
Source: MedEdPORTAL. 2020 May 27;16:10903. doi: 10.15766/mep_2374-8265.10903 (PMC7331967; doi:10.15766/mep_2374-8265.10903)
Supplement: Supplementary file 1 — Pretest.docxLecture and Notes.pptxInitial LPI Assessment.docxFinal LPI Assessment.docxPosttest.docxPre- & Posttest Answers.docx [file mep_2374-8265.10903-s001.zip › F. Pre- & Posttest Answers.docx]

1. Define the following terms according to the American Academy of Ophthalmology:

Primary Angle Closure Suspect: **≥ 180 degrees of iridotrabecular (ITC) contact.**

Primary Angle Closure**:≥ 180 ITC and elevated IOP.**

Primary Angle Closure Glaucoma**:≥ 180 ITC, elevated IOP and optic nerve damage.**

2. Which ethnic group as the highest incidence of angle closure?

a. Chinese

**b. Inuit**

c. Hispanics

d. African and African-derived

3. Benefits of using a laser iridotomy lens include all of the following EXCEPT:

a. Magnification of treatment area

b. Focuses energy on the iris

**c. Increases energy density on cornea**

d. Keeps eyelids open

4. List 4 potential complications following LPI

**Post-operative IOP spike**

**Inflammation**

**Hyphema**

**Focal cataract**

**Corneal damage or decompensation**

**Dysphotopsias**

**Glare, lines, diplopia, haloes**

**Need for repeat procedure**

5. Where should the peripheral iridotomy be placed on the iris to minimize the risk of post-laser dysphotopsia?

a. superior

b. inferior

c. nasal

**d. temporal**

6. A peripheral iridotomy should be at least what size in diameter?

**150-200 µm**
